# Distortion-Constrained Minimax Data Sanitization with Multiple Adversaries †

**DOI:** 10.3390/e28101054

**Published:** 2026-09-25

**Authors:** Amirarsalan Moatazedian, Yauhen Yakimenka, Rémi A. Chou, Jörg Kliewer

**Affiliations:** 1 Helen and John C. Hartmann Department of Electrical and Computer Engineering, New Jersey Institute of Technology, Newark, NJ 07102, USA; jkliewer@njit.edu; 2 Department of Computer Science and Engineering, The University of Texas at Arlington, Arlington, TX 76019, USA; remi.chou@uta.edu

**Keywords:** adversarial inference, Bayes estimation loss, data sanitization, heterogeneous side information, minimax optimization, privacy–utility tradeoff, release-independent estimation

## Abstract

We study a privacy-preserving data-sharing setting where a privatizer transforms private data into a sanitized release observed by an authorized reconstructor and two unauthorized adversaries, each with access to side information correlated with the private data. An adversary is considered stronger when it estimates the private data more accurately, thereby achieving a lower estimation loss. The privatizer maximizes the estimation loss of the stronger adversary while keeping the reconstruction distortion within a distortion budget. The difficulty is that the identity of the stronger adversary can vary with the distortion budget. Thus, optimizing the privatizer against a single adversary selected in advance can overlook the other adversary when that adversary estimates the private data more accurately. This motivates a constrained minimax formulation that accounts for both adversaries at every distortion budget. Penalized alternating updates train the privatizer and estimators. To evaluate the algorithm, we study finite-alphabet, scalar-Gaussian, ten-component Gaussian-mixture, and MNIST settings. For these settings, we compute theoretical reference curves under their respective assumptions to assess the learned sanitization mechanisms. We prove local conditional convergence bounds for a single-adversary recursion with fixed stochastic-gradient steps.

## 1. Introduction

### 1.1. Motivation

Distributed data sharing supports useful estimation, but each released representation can reveal information about the underlying private variable. When recipients possess different side information, the same release can support unequal estimation accuracy across recipients. Classical lossy source coding studies reconstruction under rate and distortion constraints, including decoders that already possess correlated side information [1,2]. Secure variants add secrecy, leakage, or disclosure requirements for unauthorized recipients with their own observations [3,4,5,6]. Information-theoretic sanitization similarly characterizes utility and privacy through distortion or uncertainty measures on released attributes [7]. Together, these frameworks show that privacy depends on the release mechanism, performance criterion, and each observer’s available information. Differentially private count-query mechanisms also accommodate estimators with different side information and loss functions [8].

We consider one authorized reconstructor and two noncolluding adversaries, all observing the same sanitized release. Each observer combines that release with distinct side information that is correlated with the same private variable. The optimized reconstruction distortion measures utility, while each adversary’s minimum estimation loss measures its inference capability. A lower adversarial loss means more accurate inference and, therefore, identifies the stronger adversary for that release. A distortion budget specifies the utility requirement and is enforced through the reconstruction distortion constraint. Privacy is the minimum of the two adversarial losses. An estimator is release-independent when its output ignores the sanitized release and uses only its assigned side information. We use the terms release-independent and side-information-only interchangeably. Our criterion measures expected Bayes estimation loss. Earlier source-coding and secrecy models use rate or equivocation criteria [1,3,4,5,6]. Information-bottleneck and privacy-funnel methods use mutual-information objectives [9,10]. Differential privacy instead constrains neighboring output distributions, whereas our criterion evaluates inference under a specified joint distribution [11,12].

Sharing sensor measurements provides one example. A hospital publishes a sanitized release of the heart’s electrical signal. The reconstructor also has a protected clinical measurement, while each adversary has its own side information. Estimating the signal supports clinical use but discloses confidential information. The clinical measurement remains protected when the release is copied. Adversaries may know the source distribution and all channel probabilities, which their Bayes estimators use with the sanitized release and side information. The privatizer’s random input is not supplied separately to the estimators.

The stronger adversary need not remain the same when the distortion budget changes across operating points. Changing the release channel can alter the two adversarial estimation losses unequally, which can reverse their ordering. Designing against one adversary chosen beforehand can, therefore, omit the more accurate adversary from the objective at some distortion budgets. For every candidate release channel, we compare the minimum estimation losses attainable by the two adversaries. The privatizer then maximizes this minimum at every distortion budget.

Each adversary’s expected loss averages estimation error over the joint distribution of the private variable, sanitized release and its side information. Privacy takes the smaller expectation, rather than the expectation of the smaller sample error. High-dimensional or unknown distributions can make release-channel optimization computationally or analytically impractical, motivating data-driven optimization.

Adversarial training performs sample-based optimization of parameterized estimators and sanitization mechanisms [13,14,15,16]. After training each candidate privatizer, we freeze its parameters. We initialize separate reconstructor and adversary instances from that candidate’s stored parameter states and continue estimator-only training on a disjoint data subset. This additional estimator-only training reassesses the fixed release using data separated from main training.

### 1.2. Contributions

The main contributions are as follows.

(i) We formulate distortion-constrained sanitization with two adversaries that possess different side information. The privatizer maximizes the minimum of the adversaries’ best-response losses.
(ii) For finite alphabets and Hamming loss, an equivalent mixed-integer linear program globally optimizes over all stochastic release channels on the specified fixed release alphabet. A nonsymmetric binary example exhibits a change in the stronger adversary along a specified path of optimal channels.
(iii) We derive a closed-form privacy–utility tradeoff for the scalar-Gaussian setting under a linear privatization mechanism. We also construct reference curves for a fitted Gaussian mixture model (GMM). We reuse these fitted-GMM reference curves as descriptive benchmarks for MNIST.
(iv) We develop an iterative data-driven method with separate block updates for the privatizer, reconstructor, and both adversaries while learning a sanitization mechanism from samples. We also reevaluate each fixed candidate release through additional estimator-only training.
(v) We establish local finite-iteration bounds for a single-adversary recursion conditional on containment with a stated failure probability.

A preliminary version appeared in the proceedings of IEEE ITW 2025 [17]. The conference paper considered binary and scalar-Gaussian settings, while this journal article generalizes the finite-alphabet analysis and adds Gaussian-mixture and MNIST experiments. It also adds a finite-iteration convergence analysis for a single-adversary recursion and estimator-only retraining for learned mechanisms.

### 1.3. Related Work

Wyner and Ziv characterize rate-distortion limits when a decoder possesses correlated side information unavailable to the encoder [1,2]. That framework explains how decoder knowledge improves reconstruction, but it does not optimize privacy against unauthorized estimators. Secure source-coding models add equivocation, leakage, disclosure, or reconstruction-privacy constraints for decoders with side information [3,4,5,6]. Their limits jointly involve communication rate and secrecy criteria, whereas our decision variable is the public release channel. Information-theoretic database sanitization balances distortion in released attributes against uncertainty concerning hidden private database attributes [7]. Our model shares this utility-privacy perspective but measures exposure through each adversary’s minimum achievable estimation loss.

Asoodeh et al. distinguish protection of a private variable from protection of every nonconstant measurable function of it with finite variance [18]. Such functions can represent individual attributes. Our criterion evaluates reconstruction of the complete private variable and imposes no separate requirement for each attribute.

Estimation-theoretic privacy directly evaluates an unauthorized observer through inference performance or an expected estimation-loss criterion [19,20,21]. Calmon and Fawaz, Wang and Calmon, and Wang et al. develop criteria connecting privacy with statistical estimation. Our formulation adds two adversaries with different side information correlated with the same private variable. Our formulation maximizes the minimum of the adversaries’ best-response losses for every release channel and distortion budget. Information bottleneck and privacy funnel methods regulate information retained by a representation through mutual-information objectives [9,10]. Differential privacy limits changes in an algorithm’s output distribution across neighboring datasets differing in one record [11,12]. Our criterion instead explicitly measures expected Bayes estimation loss under the specified joint distribution model. Privacy models with priors or auxiliary information show that an observer’s knowledge can alter disclosure risk [22,23,24]. We represent this dependence directly through separate variables for the reconstructor and both adversaries within one joint distribution. The identity of the stronger adversary can change as the optimized release channel changes with the distortion budget.

Classical secret sharing assigns shares so qualified coalitions recover a secret while unqualified groups remain excluded [25,26]. Access-structure source coding similarly studies recovery requirements when different user groups combine their observations securely [27]. Our main model instead releases one sanitized variable publicly and evaluates two noncolluding recipients separately. Evaluating recipients separately addresses individual inference risk rather than threshold recovery by a designated coalition.

Learned privacy mechanisms train filters or representations against adversarial inference models when analytical channel optimization is impractical [13,14,16]. Existing approaches include minimax filters, censored representations, privacy-preserving adversarial networks, and task-oriented visual encodings for data release [13,14,16,28,29]. Context-aware generative adversarial privacy also combines analytical examples with learned mechanisms under a minimax objective [15]. Our model includes an authorized reconstructor and two adversaries, each using separate side information correlated with the private variable. The distortion budget fixes the utility requirement, while the minimum adversarial loss determines the privacy objective.

Learned secure-communication systems have also considered multiple colluding or noncolluding eavesdroppers [30]. Our setting instead gives adversaries heterogeneous source-correlated side information. It optimizes their minimum Bayes estimation loss.

Studies of model inversion and privacy risk motivate additional adversary optimization when evaluating trained mechanisms [31,32,33,34,35,36]. We, therefore, freeze each candidate privatizer and retrain candidate-specific estimator instances before candidate selection.

### 1.4. Notation

Uppercase letters denote random variables, lowercase letters denote their realizations, bold symbols denote vectors or matrices, and calligraphic letters denote alphabets or parameter sets. We write E, P, Var, Cov, and tr for expectation, probability, variance, covariance, and trace, respectively. Conditional distributions are written as $P_{A|B}$.

## 2. System Model and Benchmarks

This section formulates the minimax problem and develops the benchmark models used to construct the reference curves.

### 2.1. Release, Estimators, and Objective

Let X∈X denote the private variable, and let the privatizer map *X* stochastically to a public release F∈F according to $P_{F|X}$. The authorized estimator, called the reconstructor, observes (F,Y) and produces R(F,Y). Unauthorized estimator *i*, called adversary *i*, observes $\left(F,Z_{i}\right)$ and produces $A_{i}\left(F,Z_{i}\right)$ for i∈{1,2}. Side information supplements the public release, with *Y* available to the reconstructor and $Z_{i}$ available to adversary *i*. The fixed joint distribution specifies how *Y*, $Z_{1}$, and $Z_{2}$ are correlated with *X*. Write $\hat{X}=R\left(F,Y\right)$ for the authorized estimate, and write ${\tilde{X}}_{i}=A_{i}\left(F,Z_{i}\right)$ for adversary *i*’s estimate.

Let Y and $Z_{i}$ denote the side-information spaces for the reconstructor and adversary *i*. Let ${\hat{X}}_{R}$ and ${\hat{X}}_{A}$ denote their respective decision spaces. The source, side-information, release and decision spaces are standard Borel spaces, including finite alphabets and Euclidean spaces with their Borel sets. The distortion and loss functions are measurable. These spaces admit regular conditional distributions [37] for the estimators’ posteriors. The decision rules have domains $R:F\times Y⟶{\hat{X}}_{R}$ and $A_{i}:F\times Z_{i}⟶{\hat{X}}_{A}$. The reconstruction distortion and adversarial loss functions have domains $d:X\times {\hat{X}}_{R}⟶\left[0,\infty \right]$ and $\ell :X\times {\hat{X}}_{A}⟶\left[0,\infty \right]$.

The privatizer uses only *X* and private randomness. Consequently, $F⊥(Y,Z_{1},Z_{2})|X$. For every considered release channel, define the nonempty admissible classes below.

$$\begin{array}{cc} R_{\mathrm{adm}}\; & :=\left\{R:F\times Y\to {\hat{X}}_{R}\;|\;R\;\mathrm{is}\;\mathrm{measurable}\;\mathrm{and}\;E\left[d\left(X,R\left(F,Y\right)\right)\right]<\infty \right\},\\ A_{i,\mathrm{adm}}\; & :=\left\{A_{i}:F\times Z_{i}\to {\hat{X}}_{A}\;|\;A_{i}\;\mathrm{is}\;\mathrm{measurable}\;\mathrm{and}\;E\left[\ell \left(X,A_{i}\left(F,Z_{i}\right)\right)\right]<\infty \right\}. \end{array}$$

These classes include every finite-loss Bayes rule and every finite-loss rule that ignores the sanitized release. We fix one measurable version of each displayed Bayes rule, including a deterministic tie-breaking rule for MAP estimators.

Let *D* denote the distortion budget. For a fixed release channel, the optimized reconstruction distortion is the infimum of expected distortion over admissible reconstructors. Each adversary’s best-response loss is the corresponding infimum over admissible adversarial rules.

$$\begin{array}{cc} L_{R}^{⋆}\left(P_{F|X}\right) & :=\underset{R\in R_{\mathrm{adm}}}{\inf}E\left[d(X,R(F,Y))\right],\\ L_{A_{i}}^{⋆}\left(P_{F|X}\right) & :=\underset{A_{i}\in A_{i,\mathrm{adm}}}{\inf}E\left[\ell (X,A_{i}\left(F,Z_{i}\right))\right],\;i\in \left\{1,2\right\}. \end{array}$$

All expectations use the fixed joint distribution and displayed release channel, so we omit those distributions from the function arguments. For a fixed release channel, the reconstructor minimizes expected distortion, while each adversary minimizes expected loss. The privatizer then maximizes the minimum adversarial loss subject to the reconstruction distortion constraint.

(1) $$\underset{P_{F|X}}{\sup}\min_{i\in \{1,2\}}L_{A_{i}}^{⋆}\left(P_{F|X}\right)\;\mathrm{subject}\;\mathrm{to}\;L_{R}^{⋆}\left(P_{F|X}\right)\leq D.$$

Equation (1) is the central privacy–utility optimization problem studied in this article. Larger adversarial loss means less accurate inference, so the inner minimum equals the loss of the stronger, lower-loss adversary.

Every mean-squared-error setting assumes $E{∥X∥}^{2}<\infty$. For an $n_{x}$-dimensional vector, the first loss family uses per-coordinate mean-squared error $n_{x}^{-1}{∥x-\hat{x}∥}^{2}$, whose Bayes-optimal estimator is the conditional mean. The corresponding MMSE equals the expected conditional-covariance trace divided by $n_{x}$ [38,39], and the estimators are given below.

$$R^{⋆}\left(f,y\right)=E\left[X\;|\;F=f,Y=y\right],\;A_{i}^{⋆}\left(f,z_{i}\right)=E\left[X\;|\;F=f,Z_{i}=z_{i}\right].$$

In the mean-squared-error settings, the distortion budget *D* is measured by per-coordinate MSE. For finite alphabets, the second family uses Hamming loss $1\{x\neq \hat{x}\}$, which a maximum a posteriori estimator minimizes in expectation [40,41].

$$\begin{array}{cc} R^{⋆}\left(f,y\right)\; & \in \underset{x\in X}{arg max}P\left(X=x\;|\;F=f,Y=y\right),\\ A_{i}^{⋆}\left(f,z_{i}\right)\; & \in \underset{x\in X}{arg max}P\left(X=x\;|\;F=f,Z_{i}=z_{i}\right). \end{array}$$

The displayed relations allow any MAP maximizer. When a single-valued estimator is required, fix one selection rule among tied maximizers. A randomized selector supported on the tied set gives the same conditional Hamming risk. In the finite-alphabet setting, the distortion budget *D* is the permitted Hamming-error probability.

A non-informative release has the same conditional distribution for every private source value and provides no information about *X*. Under this channel, a Bayes reconstructor uses only *Y*. The *Y*-only Bayes rule is admissible for every release channel, so optimized reconstruction distortion never exceeds its release-independent value. A non-informative release attains this release-independent value and, therefore, provides the upper endpoint.

Two adversaries suffice for switching, and each represents estimators sharing a side-information channel. For *K* noncolluding adversaries, the objective becomes

$$\underset{P_{F|X}}{\sup}\min_{1\leq i\leq K}L_{A_{i}}^{⋆}\left(P_{F|X}\right)\;\mathrm{subject}\;\mathrm{to}\;L_{R}^{⋆}\left(P_{F|X}\right)\leq D.$$

A coalition C instead observes $\left(F,{\left(Z_{i}\right)}_{i\in C}\right)$. Its Bayes loss satisfies

$$L_{C}^{⋆}\left(P_{F|X}\right)\leq \min_{i\in C}L_{A_{i}}^{⋆}\left(P_{F|X}\right),$$

because it can ignore other members’ side information. Including a coalition as another adversary requires its joint side-information distribution, which the individual channels need not determine.

**Figure 1:**
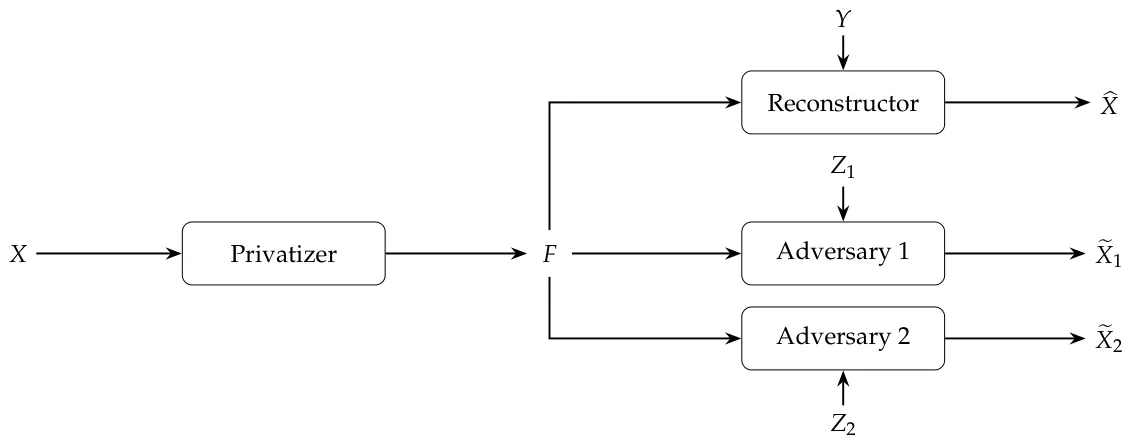
Distortion-constrained minimax data-sanitization model with two adversaries. The privatizer produces the sanitized public release *F* and maximizes the minimum of the adversaries’ best-response losses subject to the reconstruction distortion constraint. Each estimator combines *F* with its assigned side information to estimate *X*.

The noncolluding model evaluates adversaries separately without pooling their side information, as summarized in Figure 1.

### 2.2. Finite-Alphabet Benchmark

The finite-alphabet setting makes the release-channel optimization explicit. Finite alphabets represent every release probability and estimator decision with finitely many optimization variables. Let X:={0,…,M−1}, and let *X* have prior $\pi_{x}:=P\left(X=x\right)$. Fix a finite release alphabet F, and define the side-information channels below.

$$W_{Y}\left(y|x\right):=P\left(Y=y|X=x\right),\;W_{Z_{i}}\left(z_{i}|x\right):=P\left(Z_{i}=z_{i}|X=x\right).$$

For this benchmark, $F,Y,Z_{1},Z_{2}$ are conditionally independent given *X*, and each estimator applies a maximum a posteriori rule under Hamming loss. For each observation, the rule selects the source symbol with the largest posterior probability, and the resulting minimum error probability is the Bayes risk.

Let $L_{R}\left(P_{F|X}\right)$ denote the minimum reconstruction Hamming distortion, and let $L_{A_{i}}\left(P_{F|X}\right)$ denote adversary *i*’s minimum Hamming loss. Both quantities are error probabilities. Here $L_{j}\left(P_{F|X}\right)$ abbreviates the Bayes risk $L_{j}^{⋆}\left(P_{F|X}\right)$ defined above.

(2) $$\gamma^{⋆}\left(D\right):=\max_{\begin{array}{c} P_{F|X}:\\ L_{R}\left(P_{F|X}\right)\leq D \end{array}}\min\;\left\{L_{A_{1}}\left(P_{F|X}\right),L_{A_{2}}\left(P_{F|X}\right)\right\}.$$

Let $D_{\min}:=\min_{P_{F|X}}L_{R}\left(P_{F|X}\right)$ and $D_{\mathrm{SI}}:=1-\sum_{y}\max_{x}\pi_{x}W_{Y}\left(y|x\right)$ be the endpoint distortions. The subscript SI denotes the side-information-only, or release-independent, endpoint. The nontrivial tradeoff is considered for $D\in [D_{\min},D_{\mathrm{SI}}]$. The feasible set is empty when $D<D_{\min}$. For $D\geq D_{\mathrm{SI}}$, every release channel satisfies the reconstruction distortion constraint. Hence $\gamma^{⋆}\left(D\right)=\gamma^{⋆}\left(D_{\mathrm{SI}}\right)$. On every nonempty feasible set, compactness and continuity ensure that the maximum is attained. Equation (2) defines the largest achievable minimum adversarial Hamming loss at distortion budget *D*, where *D* bounds the reconstruction Hamming distortion. The release-channel probabilities $P_{F|X}\left(f|x\right)$ are the decision variables. This optimization has an equivalent mixed-integer linear formulation with the same optimal value. Appendix A gives the complete program, its equivalence proof, and the exact binary derivation. Within the fixed release alphabet, this optimization is global over all stochastic release channels under Hamming loss. This includes every deterministic map and randomized channel between the specified alphabets that satisfies the reconstruction distortion constraint.

Consider a symmetric *M*-ary crossover channel with parameter *q*, which returns the input symbol with probability 1−q. Each other symbol receives probability q/(M−1), where 0≤q≤(M−1)/M. We use a uniform source prior and symmetric channels for every observer. Here, $q_{F}$ denotes the release crossover probability, while $q_{Y}$ and $q_{Z_{i}}$ denote the side-information crossover probabilities. $L_{R}=\min\left\{q_{Y},q_{F}\right\},L_{A_{i}}=\min\left\{q_{Z_{i}},q_{F}\right\}.$ The two adversarial Hamming losses, therefore, retain one fixed ordering or remain tied throughout this symmetric family.

### 2.3. Scalar-Gaussian Benchmark

The scalar jointly Gaussian setting shows how the observers’ side-information-only conditional variances determine the tradeoff achieved by the linear privatizer below. Let $\left(X,Y,Z_{1},Z_{2}\right)$ be jointly Gaussian. The release is F=uX+vW. Here, W∼N(0,1) is independent of $\left(X,Y,Z_{1},Z_{2}\right)$. For v≠0, invertible rescaling makes the release information depend on effective signal-to-noise ratio $U:=u^{2}/v^{2}$. We use U=0 for a non-informative release. When v=0 and u≠0, the release F=uX determines *X* exactly. We represent this endpoint by U=+∞. Under squared error, the optimized reconstruction distortion and adversarial losses equal their respective conditional variances. Thus, the effective SNR ranges over $U\in {\bar{R}}_{+}:=\left[0,\infty \right]$, with the two endpoints interpreted as stated above.

**Proposition** **1** (Scalar-Gaussian tradeoff)**.** *For the scalar-Gaussian model, restrict the privatizer to F=uX+vW as defined above. Let D≥0, $V_{Y}:=\mathrm{Var}\left(X\;|\;Y\right)>0$, and $V_{Z_{i}}:=\mathrm{Var}\left(X\;|\;Z_{i}\right)>0$. Define $Q_{i}:=\left(V_{Y}-V_{Z_{i}}\right)/\left(V_{Y}V_{Z_{i}}\right)$ and $D^{\prime }:=\min\left\{D,V_{Y}\right\}$. Then*

(3) $$\max_{u,v:\;\mathrm{Var}(X\;|\;F,Y)\leq D}\min_{i\in \{1,2\}}\mathrm{Var}\left(X\;|\;F,Z_{i}\right)=\min_{i\in \{1,2\}}\frac{D^{\prime }}{1+Q_{i}D^{\prime }}.$$

*Moreover, $D^{\prime }/\left(1+Q_{i}D^{\prime }\right)\leq V_{Z_{i}}$ for each i.*

Equivalently, $Q_{i}=V_{Z_{i}}^{-1}-V_{Y}^{-1}$ compares side-information precision before the public release. Its sign identifies whether $Z_{i}$ or *Y* gives the smaller side-information-only conditional variance. Gaussian conditioning yields Proposition 1. For jointly Gaussian variables, conditional means are affine, so Bayes MMSE and linear MMSE coincide throughout this release family [38,39]. Appendix B provides the derivation of Proposition 1. Equation (3) gives the optimum within the stated jointly Gaussian model over the release family F=uX+vW. This restricted optimum can differ from the unrestricted Bayes optimum over all admissible release channels. The restriction concerns the privatizer, while the reconstructor and adversaries remain Bayes optimal over all admissible estimators. Nonlinear stochastic releases can achieve larger adversarial Bayes losses at the same reconstruction distortion.

### 2.4. Gaussian-Mixture Benchmark

A Gaussian mixture model (GMM) represents a multimodal continuous source while retaining closed-form Gaussian conditioning within each component [39,42]. A component label is said to be unobserved when its value is unavailable to the estimators. Let $X\in R^{n_{x}}$, and let the unobserved component label satisfy $C\in \{1,\ldots ,M_{C}\}$ with probability $P\left(C=c\right)=\pi_{c}$. Conditional on that label, the source distribution is given by $X\;|\;C=c∼N(\mu_{c},\Sigma_{c})$. Assume $\Sigma_{c}≻0$ for every component. The component label *C* remains hidden from the estimators, and we restrict the release to the following isotropic Gaussian family.

$$F=\alpha_{G}X+N_{F},\;N_{F}∼N(0,\sigma_{F}^{2}I_{n_{x}}).$$

The side-information channels are defined by $Y=H_{Y}X+N_{Y}$ and $Z_{i}=H_{Z_{i}}X+N_{Z_{i}}$. All Gaussian noises are mutually independent and independent of (X,C), with side-noise covariances $\Sigma_{Y}$ and $\Sigma_{Z_{i}}$. Assume $\sigma_{F}^{2}>0$, $\Sigma_{Y}≻0$, and $\Sigma_{Z_{i}}≻0$ for i∈{1,2}.

The effective release signal-to-noise ratio is defined by $U:=\alpha_{G}^{2}/\sigma_{F}^{2}$. For $\alpha_{G}\neq 0$, invertible scaling yields the equivalent release $X+U^{-1/2}\varepsilon$, where $\varepsilon ∼N(0,I_{n_{x}})$. This normalization applies only when $\alpha_{G}\neq 0$. We define U=0 separately as the non-informative endpoint. We use $U\in {\bar{R}}_{+}$ and define the endpoint U=+∞ by the limit U→∞. In this limit, $U^{-1/2}\varepsilon$ tends to zero, so the normalized release approaches X.

For the reconstructor, set $S_{R}:=Y$, $H_{R}:=H_{Y}$, and $\Sigma_{R}:=\Sigma_{Y}$. For adversary *i*, set $S_{A_{i}}:=Z_{i}$, $H_{A_{i}}:=H_{Z_{i}}$, and $\Sigma_{A_{i}}:=\Sigma_{Z_{i}}$. For $j\in \{R,A_{1},A_{2}\}$, define the stacked observation and its channel matrices below.

(4) $$\begin{array}{c} O_{j}:=\left[\begin{array}{c} F\\ S_{j} \end{array}\right],\;B_{j}:=\left[\begin{array}{c} \alpha_{G}I_{n_{x}}\\ H_{j} \end{array}\right],\;\Lambda_{j}:=\mathrm{diag}\left(\sigma_{F}^{2}I_{n_{x}},\Sigma_{j}\right),\\ \Sigma_{X|j,c}:={\left(\Sigma_{c}^{-1}+B_{j}^{T}\Lambda_{j}^{-1}B_{j}\right)}^{-1},\;\mu_{X|j,c}\left(o\right):=\Sigma_{X|j,c}\left(\Sigma_{c}^{-1}\mu_{c}+B_{j}^{T}\Lambda_{j}^{-1}o\right). \end{array}$$

Thus, conditional on C=c, $O_{j}$ is Gaussian with mean $B_{j}\mu_{c}$ and covariance $B_{j}\Sigma_{c}B_{j}^{T}+\Lambda_{j}$. Let ϕ(·;m,Γ) denote the Gaussian density with mean m and covariance Γ. Define

(5) $$\begin{array}{c} \omega_{c,j}\left(o\right):=\frac{\pi_{c}\phi \;\left(o;B_{j}\mu_{c},B_{j}\Sigma_{c}B_{j}^{T}+\Lambda_{j}\right)}{\sum_{\ell =1}^{M_{C}}\pi_{\ell }\phi \;\left(o;B_{j}\mu_{\ell },B_{j}\Sigma_{\ell }B_{j}^{T}+\Lambda_{j}\right)},\\ {\bar{\mu }}_{j}\left(o\right):=\sum_{c}\omega_{c,j}\left(o\right)\mu_{X|j,c}\left(o\right),\\ \mathrm{Var}\left(X\;|\;O_{j}=o\right):=\sum_{c}\omega_{c,j}\left(o\right)\left[\Sigma_{X|j,c}+\left(\mu_{X|j,c}\left(o\right)-{\bar{\mu }}_{j}\left(o\right)\right){\left(\mu_{X|j,c}\left(o\right)-{\bar{\mu }}_{j}\left(o\right)\right)}^{T}\right]. \end{array}$$

Equation (4) gives each component posterior. Equation (5) applies the conditional law of total covariance. Its first term averages the posterior covariance of X within each component. Its second term measures dispersion among component-specific posterior means because *C* remains unobserved.

The following Bayes-MMSE curves give the expected reconstruction distortion and adversarial losses as functions of effective release SNR.

(6) $$L_{R}\left(U\right)=E\;\left[\frac{1}{n_{x}}\mathrm{tr}\mathrm{Var}\left(X\;|\;F,Y\right)\right],\;L_{A_{i}}\left(U\right)=E\;\left[\frac{1}{n_{x}}\mathrm{tr}\mathrm{Var}\left(X\;|\;F,Z_{i}\right)\right].$$

Here $L_{j}\left(U\right)$ abbreviates the Bayes risk $L_{j}^{⋆}\left(P_{F|X}^{\left(U\right)}\right)$ of the specified Gaussian release channel. At U=0, let $L_{j}\left(0\right)$ equal the side-information-only Bayes MMSE for observer *j*. At U=+∞, set $L_{j}\left(+\infty \right)=0$. For every feasible *D*, define $U_{D}:=\min\left\{U\in {\bar{R}}_{+}:L_{R}\left(U\right)\leq D\right\}$.

**Lemma** **1** (Monotonicity of the GMM reconstruction distortion and adversarial losses)**.** *Under the stated Gaussian-mixture and channel assumptions, $L_{j}\left(U\right)$ is continuous and nonincreasing on ${\bar{R}}_{+}$ for every $j\in \{R,A_{1},A_{2}\}$. Moreover, $L_{j}\left(+\infty \right)=0$, and the minimum defining $U_{D}$ is attained for every feasible D.*

(7) $$\Gamma_{\mathrm{GMM}}\left(D\right):=\min_{i\in \{1,2\}}L_{A_{i}}\left(U_{D}\right).$$

For each effective release SNR *U*, Equations (4)–(6) determine each observer’s posterior covariance and Bayes MMSE. Lemma 1 shows that the reconstruction distortion and adversarial losses are nonincreasing in *U*. Thus, $U_{D}$ is the smallest release SNR in the stated family that satisfies the reconstruction distortion constraint. It consequently maximizes the minimum adversarial loss within that family. This argument uses monotonicity and does not require a unique maximizing SNR. Choose $U_{D}=0$ for $D\geq L_{R}\left(0\right)$ and $U_{D}=+\infty$ for D=0. At finite SNR, positive conditional covariance makes reconstruction distortion strictly positive. The functions $D\mapsto L_{A_{i}}\left(U_{D}\right)$ are the two adversarial reference curves. Equation (7) gives their lower envelope. This benchmark is family-restricted and does not assert optimality over arbitrary release channels. For fixed *U* and a realized observation, the component posterior quantities are analytic. Monte Carlo averaging approximates only the outer expectation over random observations. For an independent sample ${\left\{O_{j,k}\right\}}_{k=1}^{N}$ from the specified model, this approximation is ${\hat{L}}_{j}^{\mathrm{MC}}\left(U\right)={\left(Nn_{x}\right)}^{-1}\sum_{k=1}^{N}\mathrm{tr}\mathrm{Var}\left(X|O_{j}=O_{j,k}\right)$. The hat denotes numerical averaging. The unhatted $L_{j}\left(U\right)$ denotes the underlying expectation. Appendix B gives the derivation and proof.

### 2.5. Fitted-GMM Reference Curves for MNIST

For the MNIST assessment, we reuse the fitted-GMM reference curves as a descriptive benchmark. These curves are restricted to the fitted source model and a specified isotropic Gaussian release family. They do not establish a global optimum over arbitrary release mechanisms or a MNIST-specific optimum. The fitted-GMM reference curves use the following additive isotropic Gaussian release and side-information channels.

$$F=X+N_{F},\;Y=X+N_{Y},\;Z_{i}=X+N_{Z_{i}}.$$

All channel noises are mutually independent and independent of (X,C). Their covariances are $\sigma_{F}^{2}I_{n_{x}}$, $\sigma_{Y}^{2}I_{n_{x}}$, and $\sigma_{Z_{i}}^{2}I_{n_{x}}$. Assume $\sigma_{F}^{2}>0$, $\sigma_{Y}^{2}>0$, and $\sigma_{Z_{i}}^{2}>0$ for i∈{1,2}. Set $S_{R}:=Y$, $S_{A_{i}}:=Z_{i}$, $\sigma_{R}^{2}:=\sigma_{Y}^{2}$, and $\sigma_{A_{i}}^{2}:=\sigma_{Z_{i}}^{2}$. For $j\in \{R,A_{1},A_{2}\}$, define

(8) $$\begin{array}{c} \sigma_{\mathrm{eff},j}^{2}:={\left(\sigma_{F}^{-2}+\sigma_{j}^{-2}\right)}^{-1},\;N_{\mathrm{eff},j}∼N\left(0,\sigma_{\mathrm{eff},j}^{2}I_{n_{x}}\right),\\ T_{j}:=\sigma_{\mathrm{eff},j}^{2}\left(\frac{F}{\sigma_{F}^{2}}+\frac{S_{j}}{\sigma_{j}^{2}}\right)=X+N_{\mathrm{eff},j}. \end{array}$$

Consequently, the pair $\left(F,S_{j}\right)$ contains the same estimation information as $T_{j}$ [38,39]. Applying the Gaussian-mixture posterior formulas with Bayes-MMSE estimation gives the fitted-GMM reference curves used with the MNIST learned points. Monte Carlo averaging approximates the remaining expectation over $T_{j}$. Appendix B derives Equation (8) and records the posterior formulas.

## 3. Data-Driven Training

The preceding benchmarks optimize over all admissible estimators and over the release class specified for each benchmark. Data-driven training instead restricts those objects to parameterized models learned from finite samples. This substitution enables sample-based optimization when Bayes estimators lack tractable formulas.

### 3.1. Expected and Sample-Average Objectives

Let *p*, *r*, and $a_{i}$ parameterize the privatizer $P_{p}$ and estimators $R_{r}$ and $A_{i,a_{i}}$. Unhatted $L_{j}$ denotes expected distortion or loss for the specified estimator. A star denotes minimization over all admissible measurable estimators, and a hat denotes a sample average. Continuous training uses sample MSE. Finite-alphabet training uses differentiable classification scores, while evaluation uses hard-decision Hamming error. For a fixed privatizer *p* and adversarial parameter class $\Theta_{i}$, expected losses satisfy

(9) $$L_{A_{i}}^{⋆}\left(P_{p}\right)\leq \underset{a_{i}\in \Theta_{i}}{\inf}L_{A_{i}}\left(p,a_{i}\right)\leq L_{A_{i}}\left(p,a_{i}^{\mathrm{trained}}\right).$$

The same ordering holds for the reconstructor. Under per-coordinate squared error, conditional-mean orthogonality gives [38]

(10) $$L_{A_{i}}\left(P_{p},A\right)-L_{A_{i}}^{⋆}\left(P_{p}\right)=\frac{1}{n_{x}}E\;\left[∥A\left(F,Z_{i}\right)-E\left[X|F,Z_{i}\right]{∥}^{2}\right].$$

The trained-estimator loss, therefore, includes estimation error beyond Bayes uncertainty. Estimator-only retraining performs additional optimization within the chosen parameterized class. The privatizer remains fixed during this retraining, so the information available to each estimator is unchanged. A lower adversarial loss can, therefore, reflect improved estimation of the same release. Finite test estimates need not preserve these expected-loss inequalities.

The reconstructor updates its parameters to reduce sample-average distortion, while each adversary reduces its sample-average loss. With penalty weight ρ>0, the privatizer uses the following objective.

(11) $${\hat{L}}_{P}=-\min_{i\in \{1,2\}}{\hat{L}}_{A_{i}}+\rho {\left({\hat{L}}_{R}-D\right)}^{2}.$$

Training optimizes a parameterized finite-sample penalized surrogate of Equation (1). Trained parameterized estimators determine the reported reconstruction distortions and adversarial losses. A one-sided exterior penalty instead uses $\rho {\left[\max\left\{0,{\hat{L}}_{R}-D\right\}\right]}^{2}$ for the inequality [43].

The two-sided penalty decreases as reconstruction distortion approaches *D* for a fixed adversarial score. Learned points use achieved distortion $\hat{d}$ because finite penalized training need not satisfy $L_{R}\leq D$. The quantities $\hat{d}-D$ and ${\left[\hat{d}-D\right]}_{+}$ measure signed error and positive violation, subject to test-sample error.

Equality and inequality distortion constraints have the same supremum under the following sufficient condition. Let *d* and $b_{i}$ denote a feasible channel’s Bayes reconstruction distortion and adversarial losses, with finite side-information-only values $d_{0}$ and $b_{i0}$. Suppose $d<D\leq d_{0}$. Independently of the source and side information, select a constant release with probability $t=\left(D-d\right)/\left(d_{0}-d\right)$ and the original channel otherwise. Include a binary variable identifying the selected channel in the sanitized release. Conditioning on it gives $d_{t}=\left(1-t\right)d+td_{0}=D,\;b_{it}=\left(1-t\right)b_{i}+tb_{i0}\geq b_{i}.$ The inequality follows because each estimator can ignore the original release. Channels with d=D need no modification. Thus equality and inequality distortion constraints have the same supremum for $0\leq D\leq d_{0}$ within any class closed under this construction. The channel choice can enlarge the release alphabet, so a fixed alphabet or neural architecture need not allow this construction. For $D>d_{0}$, equality is impossible because the reconstructor can ignore the release.

A common positive ρ penalizes distortion-budget deviations in every setting. Dividing adversarial and reconstruction scores by positive scales $s_{A}$ and $s_{R}$ gives an equivalent normalized objective with weight $\lambda =\rho s_{R}^{2}/s_{A}$. Thus, scaling changes the penalty’s relative weight while preserving its purpose.

A finite penalty also gives a conditional quantitative comparison. Let m(θ) and d(θ) be the minimum adversarial score and reconstruction score for the same objective and distribution. Suppose ρ>0, ϵ≥0, a comparison state satisfies $d(\theta_{D})=D$, and $-m\left(\hat{\theta }\right)+\rho {\left(d(\hat{\theta })-D\right)}^{2}\leq -m\left(\theta_{D}\right)+\epsilon .$ If $m\left(\hat{\theta }\right)-m\left(\theta_{D}\right)\leq M$, rearrangement gives

$$\rho {\left(d(\hat{\theta })-D\right)}^{2}\leq M+\epsilon ,\;\left|d\left(\hat{\theta }\right)-D\right|\leq \sqrt{(M+\epsilon )/\rho }.$$

For genuine Hamming risks, M=1 is valid. With ρ=1000, the bound is $\sqrt{(1+\epsilon )/1000}$, or approximately 0.0316228 when ϵ=0. This is a conditional comparison, not a measured training guarantee. For fixed-distribution Bayes MSE, finite side-information-only risks bound the score range. Arbitrary trained MSE and classification surrogates require their own score bounds. An empirical comparison bounds empirical distortion. This comparison requires a state with reconstruction score *D*, the stated objective comparison, and a bound on the adversarial score increase. These hypotheses are unverified for the trained parameterizations. Their empirical distortion-budget deviations are evaluated directly in Section 5.1.

The affine reference game in Supplementary Containment Analysis, Section S1, gives a separate stationary-point calculation for the same squared-penalty design. Its stationary reconstruction distortion is D+1/(2ρ), or D+0.0005 when ρ=1000. This value concerns the specified affine game and its local assumptions in Section 4.2. It is not an upper bound for trained MLP/CNN reconstruction distortion. Finite penalization can, therefore, leave a nonzero distortion-budget deviation even at a stationary point.

During each finite-alphabet privatizer update, let *u* and *v* denote the minibatch-averaged differentiable classification-error scores from adversaries one and two. These scores are smooth training surrogates for the corresponding Hamming errors. Optimization replaces their hard minimum with the smooth expression below.

(12) $${\mathrm{smin}}_{\kappa }\left(u,v\right):=-\frac{1}{\kappa }\log\;\left(e^{-\kappa u}+e^{-\kappa v}\right),\;\kappa >0,$$

Equivalently, $0\leq \min\left\{u,v\right\}-{\mathrm{smin}}_{\kappa }\left(u,v\right)\leq \log\left(2\right)/\kappa$. Increasing κ decreases this bound, so the soft minimum approaches the hard minimum more closely.

Only finite-alphabet privatizer updates use Equation (12). Continuous privatizers retain the hard minimum, and each estimator minimizes its own score. The soft-minimum derivative averages adversarial score gradients with weights proportional to $e^{-\kappa u}$ and $e^{-\kappa v}$. At a continuous score tie, the generalized gradient is their convex hull [44]. The smoothing bound excludes classification-surrogate, optimization, and discrete-gradient approximation errors. Hard-forward and relaxed-backward surrogate gradients need not be unbiased for the hard discrete objective [45].

### 3.2. Privatizer–Estimator Updates

This subsection summarizes the data-driven procedure used to generate the learned points. Algorithm 1 updates the privatizer, reconstructor, Adversary 1, and Adversary 2 in that order. For block $b\in \{p,r,a_{1},a_{2}\}$, let $\xi_{b,t}$ contain its minibatch and fresh channel randomness at iteration *t*. Let ${\mathrm{Step}}_{b}\left(q,g\right)$ denote one step of the training optimizer for parameter block *q* using gradient *g*. Only the designated block changes during each update.

**Algorithm 1: **Data-driven training algorithm
**    Input:**
distortion budget *D*, penalty weight ρ, main-training iterations $T_{\mathrm{total}}$, and initial parameters $p,r,a_{1},a_{2}$
**    Output:**
trained parameters $p,r,a_{1},a_{2}$      1**for **$t=1,\ldots ,T_{\mathrm{total}}$** do **    Draw $\xi_{p,t}$. With $\left(r,a_{1},a_{2}\right)$ fixed, set $p\leftarrow {\mathrm{Step}}_{p}\;\left(p,\nabla_{p}{\hat{L}}_{P}\left(p,r,a_{1},a_{2};\xi_{p,t}\right)\right)$.       2Use (12) only for the finite-alphabet privatizer. Continuous privatizer updates retain the hard minimum.      3    Draw $\xi_{r,t}$. With $\left(p,a_{1},a_{2}\right)$ fixed, set $r\leftarrow {\mathrm{Step}}_{r}\;\left(r,\nabla_{r}{\hat{L}}_{R}\left(p,r;\xi_{r,t}\right)\right)$.      4    Draw $\xi_{a_{1},t}$. With $\left(p,r,a_{2}\right)$ fixed, set $a_{1}\leftarrow {\mathrm{Step}}_{a_{1}}\;\left(a_{1},\nabla_{a_{1}}{\hat{L}}_{A_{1}}\left(p,a_{1};\xi_{a_{1},t}\right)\right)$.      5    Draw $\xi_{a_{2},t}$. With $\left(p,r,a_{1}\right)$ fixed, set $a_{2}\leftarrow {\mathrm{Step}}_{a_{2}}\;\left(a_{2},\nabla_{a_{2}}{\hat{L}}_{A_{2}}\left(p,a_{2};\xi_{a_{2},t}\right)\right)$.      6
**end for**


## 4. Finite-Iteration Convergence Analysis for a Single-Adversary Recursion

For a single-adversary recursion with fixed stochastic-gradient steps, this section bounds last-iterate error and best-iterate operator residuals conditional on local containment. The assumptions specify operator regularity, conditional gradient moments and containment probability. In contrast, experiments use two adversaries and Adam with cosine learning-rate decay and weight decay. Adam also maintains running averages of gradients and their squares [46].

### 4.1. Single-Adversary Recursion

The two-adversary objective can be nonsmooth when the adversarial losses tie. We, therefore, fix one smooth adversarial loss $L_{A}\left(p,a\right)$ with parameter block *a*.

Let $p\in R^{d_{p}}$, $r\in R^{d_{r}}$, and $a\in R^{d_{a}}$ be the parameter blocks, and set θ=(p,r,a). For penalty weight ρ>0 and distortion budget *D*, define the expected objective below.

(13) $$L_{P}\left(\theta \right)=-L_{A}\left(p,a\right)+\rho {\left(L_{R}\left(p,r\right)-D\right)}^{2}$$

The game operator associated with (13) is defined below.

(14) $$G\left(\theta \right)=\left(G_{p}\left(\theta \right),G_{r}\left(\theta \right),G_{a}\left(\theta \right)\right):=\left(\nabla_{p}L_{P}\left(\theta \right),\nabla_{r}L_{R}\left(p,r\right),\nabla_{a}L_{A}\left(p,a\right)\right).$$

A zero of *G* is a stationary point of the penalized game and need not solve the original inequality-constrained problem. Let α>0 and β>0 denote the privatizer and estimator step sizes. The quantities $g_{t}^{p},g_{t}^{r},g_{t}^{a}$ estimate the operator blocks in (14). The recursion first updates the privatizer and then evaluates the reconstructor and adversary gradients at the updated privatizer.

(15) $$\begin{array}{cccc} p_{t+1} & =p_{t}-\alpha g_{t}^{p},\; & \theta_{t+\frac{1}{2}} & =(p_{t+1},r_{t},a_{t}),\\ r_{t+1} & =r_{t}-\beta g_{t}^{r}, & a_{t+1} & =a_{t}-\beta g_{t}^{a}. \end{array}$$

The estimator gradients are evaluated at $\theta_{t+1/2}$, and the resulting full state is $\theta_{t+1}=\left(p_{t+1},r_{t+1},a_{t+1}\right)$. Equation (15) writes the reconstructor and adversary updates together because neither gradient depends on the other estimator’s parameter block. They are, therefore, equivalent to updating the reconstructor before the adversary after updating the privatizer.

The proof tracks information available before and after sampling the privatizer gradient. Let $F_{t}$ include $\theta_{t}$ and all history available before sampling $g_{t}^{p}$, and define the two subsequent histories below.

$$F_{t+\frac{1}{2}}:=F_{t}\vee \sigma \left(g_{t}^{p}\right),\;F_{t+1}:=F_{t+\frac{1}{2}}\vee \sigma (g_{t}^{r},g_{t}^{a}),$$

Here, ∨ denotes the jointly generated sigma-algebra, while $\theta_{t}$ and $\theta_{t+1/2}$ are measurable with respect to $F_{t}$ and $F_{t+1/2}$, respectively. Conditional unbiasedness uses two histories because sequential updates evaluate gradients at two distinct parameter points. The sampled updates are assumed measurable and finite-valued.

At a fixed parameter state, let *ℓ* be the per-sample reconstruction loss and L=E[ℓ]. Unbiased minibatch loss and gradient averages need not have an unbiased product. For the same independent and identically distributed batch of size *B*,

$$E\left[\left(\hat{L}-D\right)\hat{\nabla L}\right]=\left(L-D\right)\nabla L+\frac{1}{B}\mathrm{Cov}\left(\ell ,\nabla \ell \right).$$

The covariance is conditional on the current state. Under i.i.d. sampling, independent batches for the two factors or products using distinct sample indices remove this bias from the penalty gradient. The theorem requires conditional unbiasedness of its specified gradient estimate.

### 4.2. Assumptions

The following assumptions state the local conditions used in the finite-iteration convergence analysis of the single-adversary recursion.

**Assumption** **1** (Local conditional unbiasedness and bounded variance)**.** *For 0≤t<T, set $J_{t}=1_{\left\{\theta_{t}\in U\right\}}$ and $J_{t+1/2}=1_{\left\{\theta_{t+1/2}\in U\right\}}$. Define the restricted gradient errors*

$$\begin{array}{cc} \xi_{t}^{p} & :=J_{t}\left(g_{t}^{p}-G_{p}\left(\theta_{t}\right)\right),\\ \xi_{t}^{b} & :=J_{t+1/2}\left(g_{t}^{b}-G_{b}\left(\theta_{t+1/2}\right)\right),\;b\in \left\{r,a\right\}, \end{array}$$

*where each expression is defined as zero when its indicator vanishes. There exists $\sigma^{2}\geq 0$ such that*

$$\begin{array}{cccc} E[\xi_{t}^{p}|F_{t}] & =0,\; & E[{∥\xi_{t}^{p}∥}^{2}|F_{t}] & \leq \sigma^{2}J_{t},\\ E[\xi_{t}^{b}|F_{t+1/2}] & =0, & E[{∥\xi_{t}^{b}∥}^{2}|F_{t+1/2}] & \leq \sigma^{2}J_{t+1/2}. \end{array}$$

*These moments are required only when the gradient evaluation state lies in U. No moment conditions outside U or conditional independence between estimator-gradient errors are assumed.*

Conditional unbiasedness and bounded conditional variance are standard in stochastic approximation and stochastic variational-inequality analysis [47,48,49,50]. The privatizer gradient is conditioned on $F_{t}$, whereas the reconstructor and adversary gradients are conditioned on $F_{t+\frac{1}{2}}$.

**Assumption** **2** (Local Lipschitz continuity and strong monotonicity)**.** *There are a closed convex set $U\subset R^{d_{p}+d_{r}+d_{a}}$, a point $\theta^{⋆}\in U$, and constants L>0 and μ>0 such that $G(\theta^{⋆})=0$. For all θ,ϑ∈U,*

$$∥G\left(\theta \right)-G\left(\vartheta \right)∥\leq L∥\theta -\vartheta ∥,\;\langle G\left(\theta \right)-G\left(\vartheta \right),\theta -\vartheta \rangle \geq \mu {∥\theta -\vartheta ∥}^{2}.$$

Lipschitz continuity and strong monotonicity are established regularity conditions in variational-inequality and stochastic-game analyses [50,51,52]. Here, $\theta^{⋆}$ is the point in U satisfying $G(\theta^{⋆})=0$. Strong monotonicity supplies descent toward $\theta^{⋆}$, while Lipschitz continuity controls operator changes between parameter evaluations. For continuously differentiable *G*, a positive-definite symmetric Jacobian at $\theta^{⋆}$ gives strong monotonicity on a sufficiently small convex neighborhood. Smoothness alone does not ensure this, and continuous parameter symmetries can create nonisolated stationary points. Supplementary Containment Analysis, Sections S1 and S2, verifies μ>0.5527 and L<1.8945 for an affine ten-component GMM game with hidden component labels. It also bounds the conditional gradient variance by $\sigma^{2}\leq 36$ for batches of 256. These constants concern its specified coordinates and gradient estimate, rather than the trained networks. Theorem 1 is applied only to the affine reference recursion. Its assumptions are unverified for the trained MLP/CNN parameterizations, so it does not cover those experiments.

**Assumption** **3** (Quantified finite-iteration containment)**.** *Fix the distortion budget D, integer horizon T≥1, step sizes, initialization and sampling rule. For 0≤t≤T, define the containment events below and assume a specified $\delta_{T}\left(D\right)\in \left[0,1\right)$ satisfies*

$$\begin{array}{c} E_{t}:=\{\theta_{j}\in U\;\left(0\leq j\leq t\right),\;\theta_{j+\frac{1}{2}}\in U\;\left(0\leq j<t\right)\},\\ \mathrm{Pr}\left(E_{T}\right)\geq 1-\delta_{T}\left(D\right). \end{array}$$

*The notation displays the dependence on D and T. The bound may also depend on the other fixed recursion parameters.*

For a reference recursion defined only until its first exit from U, keep its first outside state fixed thereafter. This convention changes neither $E_{T}$ nor the pre-exit conditional moments.

The Supplementary Containment Analysis, Sections S1–S7, gives two reference calculations with batch size 256 and horizon T=12,480, matching the ten-component GMM training count in Section 5.2. For the affine game, fix D∈[1,42.1418], $\left(\alpha ,\beta \right)\in {\left[10^{-5},10^{-3}\right]}^{2}$, $|p_{0}|\leq 0.1$ and $r_{0}=a_{0}=0$. Under the specified fresh-noise gradient estimates, every full and intermediate state remains in U={|p|≤1,∥r∥,∥a∥≤0.02} through iteration *T* with probability greater than 99.59%. Thus $\delta_{T}\left(D\right)=0.0041$ verifies Assumption 3. This guarantee concerns each separately fixed budget and step pair.

The component-conditioned scalar process in Section S7 instead uses step $10^{-4}$, 256 fresh independent component labels per update and initialization in $I_{D}$, the interval defined there from the supplied covariance spectra. All 12,481 states stay in $I_{D}$ with probability 1 for each fixed 0<D≤27.9449 and with probability at least 0.99 for each fixed 27.9449<D≤38.7477. For $38.7477<D<{\bar{v}}_{0}\approx 42.1628$, Section S7 gives the explicit lower bound and its 256-point figure, where ${\bar{v}}_{0}$ is the component-conditioned side-information-only distortion. A zero lower bound does not establish failure. The affine lower endpoint ensures positive release-noise variance throughout its neighborhood, while the distinct scalar calculation also covers 0<D<1. These probabilities concern the specified reference processes and do not estimate neural Adam convergence.

### 4.3. Finite-Iteration Convergence Bounds

The following theorem gives the main finite-iteration convergence bounds for the single-adversary recursion under the preceding assumptions.

**Theorem** **1** (Finite-iteration convergence bounds for a single-adversary recursion)**.** *For recursion (15) with α,β>0, let $\theta_{0}\in U$ be deterministic and impose Assumptions 1–3. Write $\theta^{⋆}=\left(p^{⋆},r^{⋆},a^{⋆}\right)$ and set $\delta =\delta_{T}\left(D\right)$. Define the constants and weighted error below.*

$$\begin{array}{c} \begin{array}{cccc} \eta & :=\min\{\alpha ,\beta \},\; & C_{\alpha ,\beta } & :=\alpha +2\beta +4L^{2}\alpha^{2}\left(\beta +\frac{2}{\mu }\right),\\ \lambda & :=\frac{7\mu }{4L^{2}}-C_{\alpha ,\beta }, & c & :=\min\{1,\lambda L^{2}\eta \}, \end{array}\\ V_{t}:=\alpha^{-1}{∥p_{t}-p^{⋆}∥}^{2}+\beta^{-1}\left({∥r_{t}-r^{⋆}∥}^{2}+{∥a_{t}-a^{⋆}∥}^{2}\right). \end{array}$$

*If $C_{\alpha ,\beta }<7\mu /\left(4L^{2}\right)$, then, for 0≤t≤T and 1≤s≤T,*

(16) $$\begin{array}{c} E\left[V_{t}|E_{T}\right]\leq \frac{{\left(1-c\right)}^{t}V_{0}+C_{\alpha ,\beta }\sigma^{2}/c}{1-\delta }, \end{array}$$

(17) $$\begin{array}{c} \min_{0\leq t<s}E\;\left[{∥G\left(\theta_{t}\right)∥}^{2}|E_{T}\right]\leq \frac{V_{0}/\left(\lambda s\right)+C_{\alpha ,\beta }\sigma^{2}/\lambda }{1-\delta }. \end{array}$$

The step condition gives λ>0 and contraction factor 1−c for the weighted squared distance $V_{t}$. Equations (16) and (17) bound the conditional last-iterate error and the smallest conditional expected squared operator norm. The proof first bounds moments weighted by current-containment indicators, then divides by $\mathrm{Pr}\left(E_{T}\right)\geq 1-\delta_{T}\left(D\right)$. Thus oracle identities never condition on future containment. When $\delta_{T}\left(D\right)=0$, the bounds are unconditional. The event $E_{T}$ requires every full and intermediate state through iteration *T* to remain in U. A smaller exit-probability bound reduces the factor $1/(1-\delta_{T}\left(D\right))$ but does not eliminate the gradient-noise terms.

With exact population gradients and deterministic initialization, $\sigma^{2}=0$ and Assumption 3 implies certain containment, so one may take $\delta_{T}\left(D\right)=0$. Containment and contraction rate remain distinct, as the low-distortion scalar calculation in Supplementary Containment Analysis, Section S7, shows. Appendix C gives the complete proof, including the comparison update used to handle intermediate exits.

## 5. Numerical Results

The numerical study assesses learned points against model-derived reference curves in four settings. A reference curve maps reconstruction distortion to adversarial loss under a specified source model, side-information channels, and admissible release family. A reference comparison overlays learned test points, plotted at achieved reconstruction distortions, on the applicable reference curves. Each learned point reports its test reconstruction distortion and adversarial losses after estimator retraining.

The finite-alphabet curve is globally optimal over all stochastic channels on the specified binary release alphabet. The scalar-Gaussian reference curves are exact for jointly Gaussian variables within the stated linear privatizer family. The fitted-GMM reference curves use Bayes-optimal estimators under the fitted source model and specified Gaussian channels. For that model, the effective release SNR chosen at each distortion budget maximizes the minimum adversarial loss only within the specified isotropic Gaussian release family. They do not establish global optimality over arbitrary release mechanisms.

### 5.1. Data, Architectures, and Training Configuration

The learned-mechanism experiments use four disjoint data subsets. They support main training, estimator retraining, validation-based selection, and final test evaluation, respectively. The retraining–validation pool contains 20% as many samples as the main-training subset. We allocate 75% of this pool to estimator retraining and 25% to validation.

The finite-alphabet privatizer maps a one-hot source through one 2048-unit ReLU layer to two release logits. Each finite-alphabet estimator uses the same hidden width with concatenated one-hot release and side-information inputs. In the scalar-Gaussian experiment, the privatizer receives *X* and fresh standard-Gaussian private randomness $W_{P}∼N\left(0,1\right)$ as its two inputs. The random input is independent of $\left(X,Y,Z_{1},Z_{2}\right)$. The reconstructor and each adversary receive the release and their assigned side information. All four networks use 2→50→1 multilayer perceptrons.

No scalar-Gaussian layer uses an activation. The privatizer, therefore, has the affine form $F=uX+vW_{P}+b$. Removing the deterministic offset *b* preserves every estimator’s information. This matches the release family in Proposition 1, with affine Bayes estimators. Nonlinear activations would extend the release class beyond that reference.

For the ten-component GMM and MNIST experiments, the privatizer concatenates X with fresh private randomness $W_{P}$ of the same shape. Its entries are i.i.d. $N(0,16^{2})$ variables and are independent of the data and side-information samples. The resulting two-channel tensor enters the convolutional privatizer, which outputs the one-channel sanitized release F. The reconstructor and each adversary concatenate F with their assigned side information. The privatizer, reconstructor, and both adversaries use the same convolutional architecture. Each of these four networks begins with a stride-two 3×3 convolution containing 32 channels. Batch normalization and a leaky rectifier with slope 0.2 follow, while a matching transpose convolution completes the network [53,54].

The ten-component GMM experiment instead evaluates nonlinear learned releases under a specified multimodal source. The Gaussian-release comparison for the ten-component GMM is descriptive only. No matched-family GMM comparison is reported. MNIST reuses that fitted-source reference descriptively.

Main training uses Adam with batch size 256 for 64 epochs [55]. The main-training learning rate is $10^{-3}$ for the privatizer, reconstructor, and both adversaries in every setting. Cosine learning-rate annealing reduces it from $10^{-3}$ to $10^{-5}$ [56]. Every block update draws fresh side information and release randomness. Continuous runs use mixed-precision arithmetic [57]. Weight decay is $10^{-5}$, and the penalty weight is ρ=1000. Section 3.1 gives its loss-scale interpretation. The squared penalty in Equation (11) is the only mechanism that encourages reconstruction scores toward the assigned distortion budget. Neither projection nor checkpoint screening enforces agreement with that budget. Deviations can, therefore, vary across independently trained releases. Only ρ=1000 is evaluated, so the experiments do not assess sensitivity to the penalty weight. Its relative effect also depends on the loss scales described in Section 3.1.

Each setting uses 30 equally spaced distortion budgets. The finite-alphabet range is 0.01–0.2225, and the scalar-Gaussian range is 0.01–5.6925207756. Their upper limits are the respective side-information-only reconstruction endpoints, with the scalar value rounded here. The ten-component GMM and MNIST share the range 1.45–42.14178848. This upper limit is the Monte Carlo estimate of the fitted-GMM side-information-only reconstruction distortion. MNIST reuses this fitted-model budget range. This range does not identify the MNIST Bayes endpoint.

The file sanitizationexperimentplotcoordinates.csv records assigned budgets, achieved reconstruction distortions, both adversarial losses, signed deviations $\hat{d}-D$, and positive violations ${\left[\hat{d}-D\right]}_{+}$. These quantities use test reconstruction distortion after estimator-only retraining with the selected privatizer fixed. A finite squared penalty encourages proximity to the assigned budget without enforcing exact agreement. Signed deviations can, therefore, be positive or negative. Their pointwise values are recorded in this file.

A positive violation identifies an empirical exceedance of the assigned budget. The achieved reconstruction distortion and adversarial losses still describe the evaluated mechanism. Such a point is displayed at $\hat{d}$ and is not reported as feasible at *D*. A negative signed deviation lies below the assigned budget. For finite alphabets, the deviations use test Hamming error, whereas the training penalty uses a differentiable classification score. These comparisons describe empirical constraint satisfaction and remain subject to test-sample error.

The privatizer and estimators share an architectural template but learn separate parameters for their respective objectives. Shared architecture does not imply equal approximation accuracy. Wider activation-free scalar networks retain the same affine function class.

The finite-alphabet privatizer uses a hard Gumbel–Softmax release [58,59]. Its forward pass is a discrete one-hot vector, while a differentiable relaxation supplies gradients. The Gumbel–Softmax temperature τ controls the concentration of that relaxation. Smaller τ makes the relaxed sample closer to a one-hot vector. Estimator updates use τ=1. During privatizer updates, τ decreases from 0.5 to 0.01 as main training proceeds. This decrease brings the differentiable relaxation progressively closer to the discrete forward output.

For fixed logits, positive temperature preserves the hard Gumbel maximizing index and release distribution while changing the gradient relaxation. The relaxed-softmax Jacobian contains 1/τ and can amplify gradients near ties. Only the finite-alphabet privatizer objective uses the soft minimum in (12) with κ=12. Its worst-case smoothing gap is log(2)/12≈0.058 in surrogate-score units. Plotted values use ordinary Hamming error. Because the penalty is two-sided, learned points are plotted at their achieved reconstruction distortions rather than their assigned distortion budgets.

Table 1 records the sample counts, learning rates, numbers of distortion budgets, and reference-construction grid sizes. For finite-alphabet and scalar-Gaussian settings, Grid gives the reference-construction grid size. For the ten-component GMM setting, Grid gives the number of effective release-SNR values in the initial sweep. The MNIST row repeats that value because it uses the grid for the fitted-GMM reference curves. These construction grids differ from both the 30 training budgets and the retained plotting coordinates. Each learned point requires separate training and estimator retraining, whereas denser reference sampling improves the resolution of the comparison curves.

### 5.2. Checkpoint Selection and Evaluation Protocol

One main-training iteration completes the four ordered block updates in Algorithm 1. Each nominal epoch randomly permutes the active training subset and processes disjoint batches of B=256 samples. Any incomplete remainder is discarded. For $N_{\mathrm{main}}$ main-training samples, one epoch contains $T_{\mathrm{epoch}}:=\left\lfloor N_{\mathrm{main}}/B\right\rfloor$ iterations. For $E_{\mathrm{total}}=64$ epochs, $T_{\mathrm{total}}=E_{\mathrm{total}}T_{\mathrm{epoch}}$. This gives 2496 main-training iterations for the finite-alphabet and scalar-Gaussian settings and 12,480 for the ten-component GMM and MNIST. The final three epochs, therefore, contain $3T_{\mathrm{epoch}}$ iterations. Eight scheduled completed iterations from each final epoch are saved, yielding at most 24 checkpoints per distortion budget. Each checkpoint stores the post-update parameter states $\left(p,r,a_{1},a_{2}\right)$. Each checkpoint defines one candidate release mechanism through its stored privatizer state. Candidate screening is the preliminary estimator-retraining and validation stage applied to every candidate for one distortion budget. For each candidate, copies of its stored reconstructor and adversary parameter states initialize one screening reconstructor and two screening adversaries. The candidate privatizer remains fixed while these screening estimators train for eight epochs on 25% of the retraining subset. Validation scores each candidate by the minimum of its two adversarial losses. The three highest-scoring candidates advance. Their screening estimators continue training for 32 additional epochs on the entire estimator-retraining subset. This subset includes the quarter used during initial screening. Validation reevaluation then selects the highest-scoring candidate.

An exact tie in the final validation criterion is resolved by selecting the checkpoint stored earlier during main training. The test subset is not used for candidate selection. After selection, all screening estimators are discarded. One final reconstructor and two final adversaries are initialized from the corresponding parameter states in the selected checkpoint. The selected privatizer remains fixed while these estimators train for 256 epochs on the entire estimator-retraining subset at learning rate $10^{-4}$. The 32-epoch continuation and final 256-epoch retraining stages draw complete batches from the full estimator-retraining subset using the same epoch rule. This subset contains 1500 samples for the finite-alphabet and scalar-Gaussian settings and 7500 for the ten-component GMM and MNIST. The reported reconstruction distortion and each adversarial loss are averaged over three fresh stochastic forward passes. At fixed trained parameters, these passes vary only evaluation randomness and do not measure variation across independent training runs.

Checkpoint counts and retraining durations balance computation with reduced loss fluctuations, based on observed training behavior without fitting theoretical curves. Screening reserves longer training for promising candidates but ranks adversarial scores without testing budget feasibility. Figures, therefore, use achieved test reconstruction distortion. Finite-sample selection can produce optimistic validation scores [60]. Stagewise checkpoint-change frequencies and sensitivity to stage lengths or screening rules are not reported. Accordingly, the present experiments do not quantify selection bias.

For *M* fixed candidate–estimator configurations, let $s_{k}=\min_{i\in \{1,2\}}E\left[\ell_{ki}\right]$ and ${\hat{s}}_{k}$ be its validation estimate. If $|{\hat{s}}_{k}-s_{k}|\leq \epsilon$ for all *k*, their respective maximizers $k^{⋆}$ and $\hat{k}$ satisfy

$$s_{\hat{k}}\geq {\hat{s}}_{\hat{k}}-\epsilon \geq {\hat{s}}_{k^{⋆}}-\epsilon \geq s_{k^{⋆}}-2\epsilon =\max_{k}s_{k}-2\epsilon .$$

Suppose the configurations are fixed independently of *n* independent validation observations and both adversarial losses lie in [0,1]. The minimum is one-Lipschitz in the maximum norm. Hoeffding’s inequality [61] and a union bound over the 2M losses give $\epsilon =\sqrt{\frac{\log(4M/\delta )}{2n}},0<\delta <1,$ with simultaneous probability at least 1−δ, even when candidates share validation observations. Unbounded MSE, validation-dependent fitting and successive screening require separate conditions. It does not certify nominal-budget feasibility or candidates outside the comparison set.

The construction of the fitted-GMM reference curves begins with a 50-point effective-release-SNR sweep. Each SNR uses 16,000 simulated observations. Separately, bisection selects an SNR for each of 30 distortion budgets. Each provisional SNR is evaluated with 4000 observations. The search stops when its SNR interval satisfies the $10^{-5}$ tolerance. This tolerance controls the SNR interval rather than Monte Carlo error or feasibility at the assigned budget. A final 64,000-sample evaluation provides the reference reconstruction distortion and adversarial losses at each selected SNR.

**Remark** **1** (Separation of learning and benchmarking)**.** *Training and checkpoint selection use only samples and empirical objectives. Reference-curve values, benchmark release channels, and analytic Bayes rules do not enter gradients or checkpoint scores. No monotonicity or cross-budget ordering is imposed. A separate model is trained and selected for each distortion budget. Benchmark calculations construct the reported reference curves and delimit the distortion-budget ranges only for plotting and comparison. We restrict simulated budgets to intervals covered by the corresponding theory so every learned point can be compared with a theoretical reference curve. Without that comparison purpose, the simulations could cover a wider range, including distortion budgets for which no theoretical reference curve is reported.*

### 5.3. Finite-Alphabet Binary Setting

This experiment compares learned points against the exact binary reference curve and the adversarial Hamming losses along the selected optimal-channel path.

The reported instance uses X=F={0,1}, source prior $\left(\pi_{0},\pi_{1}\right)=\left(0.575,0.425\right)$, and

$$W_{Y}=\left(\begin{array}{cc} 0.65 & 0.35\\ 0.05 & 0.95 \end{array}\right),\;W_{Z_{1}}=\left(\begin{array}{cc} 0.925 & 0.075\\ 0.45 & 0.55 \end{array}\right),\;W_{Z_{2}}=\left(\begin{array}{cc} 0.525 & 0.475\\ 0.1 & 0.9 \end{array}\right).$$

The displayed prior and channel probabilities are terminating decimals, so exact enumeration treats them as rational numbers with powers of ten as denominators. Rows in these matrices index the private symbol *x*. Columns index the observed side-information symbol. The exact reference curve has five adjacent distortion-budget intervals on which its value is affine. Multiple release channels can attain the same optimal value. A fixed deterministic tie-breaking rule selects one representative optimal channel at each distortion budget. These representatives form the selected optimal-channel path.

**Figure 2:**
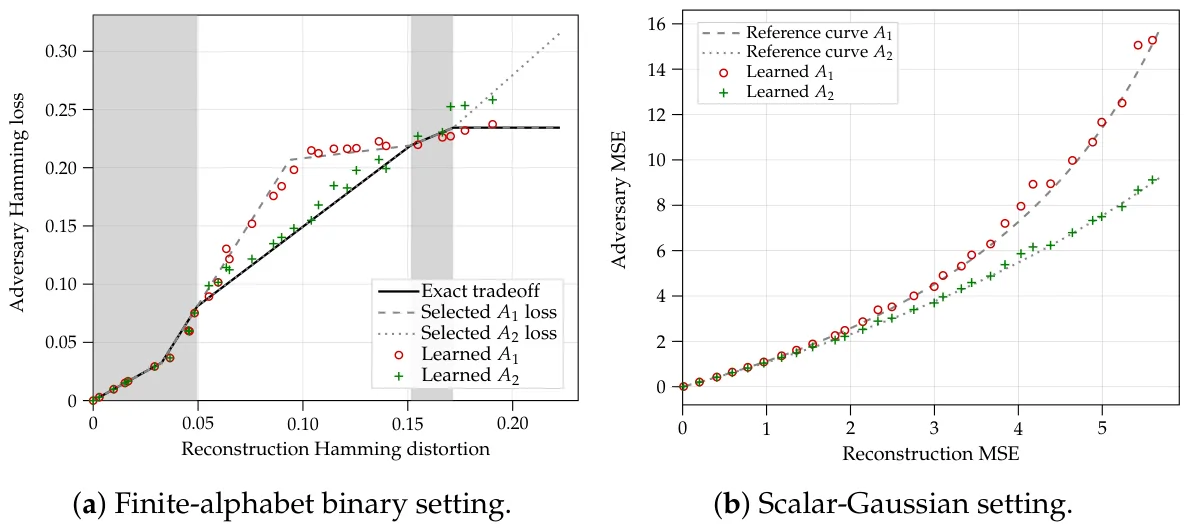
Finite-alphabet and scalar-Gaussian results. Both subfigures show reference curves and learned test points. Panel (**b**) matches the affine release family but uses trained estimators. Subfigure (**a**) also shows the adversarial Hamming losses along the selected optimal-channel path and shades both tie intervals. Learned markers use achieved test reconstruction distortion on the horizontal axis and adversarial test loss on the vertical axis. Panel (**a**) uses Hamming loss, whereas panel (**b**) uses mean squared error. The shaded intervals identify equal adversarial losses along the selected optimal-channel path. The solid curve remains the exact binary optimum.

The selection maximizes the minimum adversarial loss, followed by their sum, and then reconstruction distortion. Equation (A10) fixes the release-label choice. At D=1/10 every optimal binary channel has $L_{A_{1}}-L_{A_{2}}=47/800$. At D=9/50 primary optimizers allow differences in [−207/15520,0]. The secondary criterion selects −207/15520. The dashed and dotted curves in Figure 2a report the adversarial Hamming losses attained by the MAP rules along this path. They coincide for 0≤D≤0.0496 and 0.1514≤D≤0.1717. Both tie ranges are shaded for better visibility.

At D=0.1, Adversary 2 has the smaller loss along the selected path. At D=0.18, Adversary 1 has the smaller loss. Thus, the stronger adversary changes along this representative optimal-channel path. This proves existence for the stated model and selection rule. It makes no prevalence claim across models or tied optimizers. This switching statement concerns the path selected by the tie-breaking rule. The solid reference curve remains the exact global optimum over the specified binary release channels independently of that selection. The learned markers report adversarial test Hamming losses at their achieved reconstruction Hamming distortions.

### 5.4. Scalar-Gaussian Setting

The scalar-Gaussian experiment draws X∼N(4,16) and generates $S=b_{S}+c_{S}X+\varepsilon_{S},S\in \left\{Y,Z_{1},Z_{2}\right\},$ where the residuals are zero-mean Gaussian, mutually independent, and independent of *X*. The coefficients follow a moment-matching parametrization calculated from each target marginal mean, variance, and covariance using the displayed equations.

$$c_{S}=\frac{k_{S}}{16},\;b_{S}=m_{S}-4c_{S},\;\mathrm{Var}\left(\varepsilon_{S}\right)=v_{S}-16c_{S}^{2}.$$

Table 2 lists the sampler coefficients obtained from marginal means (3,4.5,5), marginal standard deviations (0.95,3.5,1.5), and covariances with *X*(3.05,1.5,3.9) for $\left(Y,Z_{1},Z_{2}\right)$. Displayed coefficients are rounded to at most four decimal places, while the defining moments determine the unrounded values.

Under the stated independent-residual coupling, $\mathrm{Cov}(Y,Z_{1})=0.2859$, $\mathrm{Cov}(Y,Z_{2})=0.7434$, $\mathrm{Cov}(Z_{1},Z_{2})=0.3656$. The resulting release-independent conditional variances are

$$V_{Y}=\mathrm{Var}\left(X\;|\;Y\right)\approx 5.6925,\;V_{Z_{1}}=\mathrm{Var}\left(X\;|\;Z_{1}\right)\approx 15.8163,\;V_{Z_{2}}=\mathrm{Var}\left(X\;|\;Z_{2}\right)=9.24.$$

These conditional variances rank the release-independent reconstruction distortion and adversarial estimation losses. The side observation *Y* is most informative, and $Z_{2}$ is more informative than $Z_{1}$.

The 30 distortion budgets range from 0.01 to Var(X|Y). Figure 2b compares retrained test points against the scalar-Gaussian reference curves from Proposition 1. Under its assumptions, these curves are optimal among privatizers of the form F=uX+vW.

Adversary 2 has the lower learned loss, consistent with the release-independent conditional variances. Both learned adversarial losses generally increase with achieved reconstruction distortion. Neither for the scalar-Gaussian setting nor for the GMM were trained estimators evaluated against exact Bayes estimators for their learned releases. Gaussian-channel posterior formulas do not directly apply to nonlinear learned GMM releases.

### 5.5. Ten-Component Gaussian-Mixture Setting

Before running the synthetic experiment, we estimated source parameters from all 70,000 rows of the public MNIST dataset mnist_784. OpenML hosts this dataset as data ID 554 [62,63,64]. Each MNIST image contains 28×28 pixels, so its vectorized representation has 784 coordinates. Each digit class supplied one full-covariance Gaussian component fitted to its corresponding dataset rows on the original pixel scale. Digit labels determine which images are used to estimate each Gaussian component. We add $10^{-2}I_{784}$ to every empirical covariance. This addition makes each fitted covariance positive definite and stabilizes the inversions used in posterior calculations. Empirical digit frequencies define the mixture weights because the class counts are unequal. We draw independent vectors from the resulting ten-component mixture in the 784-dimensional pixel space, forming the synthetic dataset evaluated here.

The specified side-information noise variances are $\sigma_{Y}^{2}=96$, $\sigma_{Z_{1}}^{2}=200$, and $\sigma_{Z_{2}}^{2}=72$. Thus, $Z_{1}$ is the noisiest side-information channel, *Y* is intermediate, and $Z_{2}$ is the least noisy. These variances define the two fitted-GMM reference curves.

**Figure 3:**
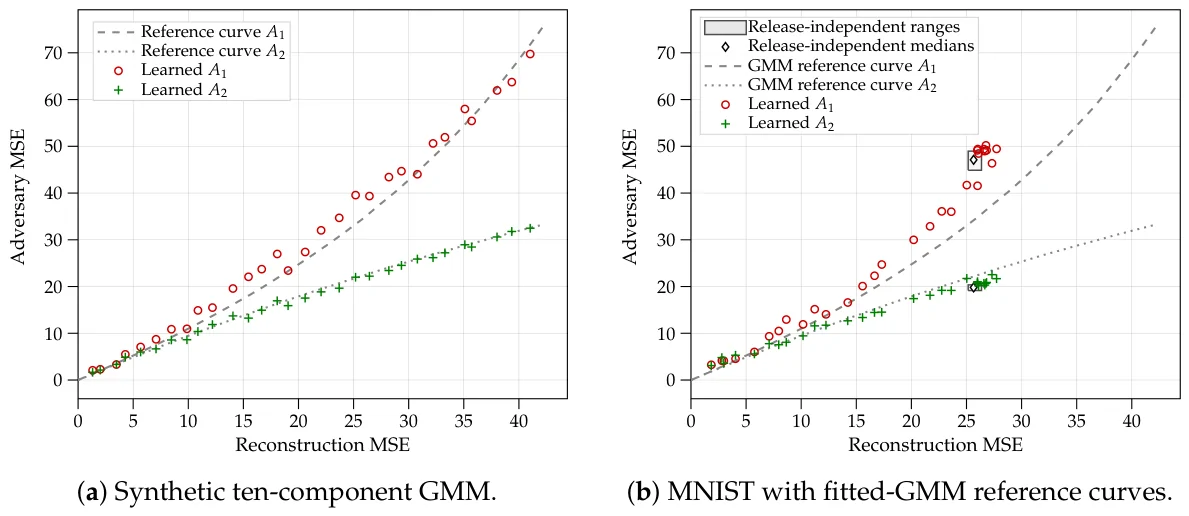
Ten-component GMM and MNIST results. Both panels compare nonlinear learned releases descriptively with restricted Gaussian-release reference curves. They are not used to assess unrestricted optimality or Bayes estimation accuracy. Subfigure (**b**) reuses those fitted-GMM reference curves descriptively for MNIST and includes separate side-information-only diagnostics. Points use achieved test reconstruction distortion. Rectangles combine separate observed coordinate ranges and are not confidence regions. Their corners and coordinatewise medians need not represent individual runs. Both axes report per-coordinate MSE for learned points and reference curves.

We evaluate the fitted-GMM reference curves at 50 effective release-SNR values. At each SNR and simulated observation, analytic posterior formulas determine every observer’s conditional covariance. Averaging its per-coordinate trace approximates the outer expectation defining Bayes MSE in Equation (6). At zero release SNR, each Bayes estimator relies only on its assigned side information. All three mean squared errors then reach their side-information-only values. The privatizer cannot increase these Bayes MSEs because each estimator can ignore the release. Neither reconstruction distortion nor adversarial loss increases further. The reference curves, therefore, terminate at the side-information-only endpoint when plotted against achieved reconstruction distortion. Monte Carlo averaging estimates this endpoint for the fitted GMM. Figure 3a compares ten-component GMM learned points against these curves.

The ten-component GMM learned adversarial losses generally increase with achieved reconstruction distortion and usually follow the ordering of the fitted-GMM reference curves.

### 5.6. MNIST Setting

The MNIST experiment uses the same mnist_784 dataset. It follows the standard 60,000/10,000 training and test partition [64]. Each image contains 28×28 pixels. Vectorization produces 784 coordinates on the original 0–255 scale. The training partition supplies 50,000 main-training images, 7500 estimator-retraining images, and 2500 validation images. The test subset does not influence checkpoint selection. Reported MNIST reconstruction distortions and adversarial losses are obtained after estimator-only retraining with the selected privatizer fixed.

Figure 3b reuses the fitted-GMM reference curves from the ten-component GMM comparison in Figure 3a. These curves retain the same fitted source, isotropic release family, and fixed side-information channels. This reuse is descriptive and does not establish a MNIST-specific optimum.

The reference source was fitted to all 70,000 images including the standard test images. For MNIST, the fitted-source calculations provide descriptive reference curves and the distortion-budget ranges used for comparison. Reference-curve values do not enter training losses or gradients, checkpoint scores, or estimator retraining.

The learned MNIST adversarial losses generally increase with achieved reconstruction distortion and broadly follow the ordering indicated by the descriptive fitted-GMM reference curves.

Figure 3b also reports release-independent estimates from 25 separate estimator-training runs. In these runs, the release carries no information about *X*. Each observer, therefore, estimates *X* from its assigned side information alone. Each outlined rectangle combines the reconstruction-MSE range horizontally with the corresponding adversarial-MSE range vertically. The corresponding diamond gives the coordinatewise medians. The reconstruction MSE lies in [25.1556,26.3629]. The Adversary 1 and Adversary 2 MSEs lie in [44.8728,48.9665] and [19.1401,20.3629], respectively. Their median points are (25.6459,47.1189) and (25.6459,19.8067). These summaries quantify run-to-run variation when estimation uses side information alone. They also help interpret the learned cluster near reconstruction MSE 25–27. For the MNIST source and specified side-information model, let $D_{\mathrm{SI}}^{R}$ and $B_{i}^{\mathrm{SI}}$ denote the side-information-only Bayes distortion and losses. Every release satisfies $L_{A_{i}}^{⋆}\leq B_{i}^{\mathrm{SI}}$ because adversaries can ignore it. For $D\geq D_{\mathrm{SI}}^{R}$, an independent release is feasible and attains these bounds. The unrestricted Bayes tradeoff for this MNIST source, therefore, saturates at $\min_{i}B_{i}^{\mathrm{SI}}$. The observed cluster is consistent with saturation. Its trained-estimator medians do not identify the Bayes endpoint. They are not endpoints of the fitted-GMM reference curves because those reference curves use the fitted Gaussian-mixture source instead of the MNIST source. The cluster is, therefore, interpreted descriptively rather than as evidence of release independence. The present experiments do not isolate the training and selection factors responsible for it or the contrast with Figure 3a.

Pixelwise MSE assesses reconstruction privacy. It does not bound digit-label disclosure or another semantic inference task [33].

### 5.7. Interpretation Across Settings

For the scalar-Gaussian, ten-component GMM and MNIST side-information channels, Adversary 2 can generate Adversary 1’s side information by adding independent Gaussian noise after invertible scaling where needed. The added variance is 200−72=128 per coordinate for the ten-component GMM and MNIST. It can retain the public sanitized release and apply any estimator used by Adversary 1 to the generated side information, reproducing its expected loss. Thus Adversary 2’s Bayes loss is no larger for any admissible release [65] and determines the Bayes minimum throughout the distortion range. Trained-estimator losses need not share this ordering. No single-adversary-trained baseline is reported for these three continuous settings, and no empirical advantage of two-adversary training is claimed.

Further adversary optimization can lower an adversarial loss, whereas further privatizer optimization can raise it. Underoptimization in either block changes the optimization problem faced by the other block. Finite minibatches can produce gradient fluctuations near stationary points. Parameter and measured-loss changes need not imply sustained population-objective improvement or systematic underoptimization.

Finite data, minibatch randomness, finite training, parameterized architectures, penalty enforcement, checkpoint selection, and restricted release families can all create gaps. The isotropic Gaussian release restriction affects the fitted-GMM and MNIST comparisons. For MNIST, source-model mismatch is an additional source of learned–reference disagreement. Consequently, no individual gap identifies a unique cause. A learned point above a reference curve does not by itself establish that its release mechanism improves on the corresponding benchmark mechanism.

## 6. Conclusions

We studied privacy-preserving data sharing with one authorized reconstructor and two noncolluding adversaries. Each observer combines a common sanitized release with side information correlated with the private data. Our constrained minimax formulation maximizes the minimum of the adversaries’ best-response losses subject to the reconstruction distortion constraint at each distortion budget. It evaluates both adversaries at every distortion budget because the lower-loss adversary can change with the distortion budget.

For finite alphabets, an equivalent mixed-integer linear program globally optimizes over all stochastic release channels on the specified fixed release alphabet. The exact solution for the specified nonsymmetric binary model exhibits switching along a selected tie-broken path of optimal channels. The adversarial Hamming losses also coincide over explicit distortion-budget intervals. The scalar-Gaussian result gives a closed-form restricted optimum over the linear release family F=uX+vW. The GMM and MNIST studies extend the assessment to multimodal continuous sources and image data.

The iterative data-driven method learns sanitization mechanisms through ordered privatizer, reconstructor, and adversary block updates. Estimator-only retraining then reevaluates each selected release using separately retrained reconstructor and adversary instances. Across all four settings, larger achieved reconstruction distortion generally accompanies larger learned adversarial losses. These comparisons assess the learned mechanisms against reference curves derived under explicit model assumptions.

The single-adversary analysis gives conditional local finite-iteration bounds for fixed stochastic-gradient steps under quantified containment.

## Figures and Tables

**Table 1:** Settings used for the reported numerical results. Main training uses cosine annealing from $10^{-3}$ to $10^{-5}$ over 64 epochs. The final 256 estimator-only epochs use learning rate $10^{-4}$.

Setting	Main	Retrain	Validation	Test	Initial Learning Rate	Distortion Budgets	Grid
Finite alphabet	10,000	1500	500	2000	$10^{-3}$	30	2048
Scalar-Gaussian	10,000	1500	500	2000	$10^{-3}$	30	2048
Ten-component GMM	50,000	7500	2500	10,000	$10^{-3}$	30	50
MNIST	50,000	7500	2500	10,000	$10^{-3}$	30	50

**Table 2:** Affine scalar-Gaussian sampler. Displayed coefficients are rounded to at most four digits after the decimal point.

*S*	$b_{S}$	$c_{S}$	$\mathrm{Var}(\varepsilon_{S})$
*Y*	2.2375	0.1906	0.3211
$Z_{1}$	4.125	0.0938	12.1094
$Z_{2}$	4.025	0.2438	1.2994

## Data Availability

The MNIST data are publicly available as OpenML mnist_784, data ID 554 and version 1, at https://www.openml.org/d/554 (accessed on 17 August 2026). The article reports the distributions and numerical parameters used for the finite-alphabet and scalar-Gaussian results. The article’s Supplementary Materials provide verification code, fitted-source parameters, numerical certificates and plot coordinates.

## Supplementary Materials

The following supporting information can be downloaded at https://www.mdpi.com/article/10.3390/e28101054/s1. The document Supplementary Containment Analysis gives the complete proofs, numerical evaluation and containment figure for Section 4.2. The files in SupportingFiles/Manuscript provide executable verification, source parameters and plot coordinates. The program finitealphabetexactbounds.py and its JSON and CSV certificates verify Appendix A. The file mnisttencomponentgmmparameters.npz and its readable CSV copies specify the fitted source. The affine and spectral containment programs use the accompanying source tables. The file sanitizationexperimentplotcoordinates.csv records all plotted coordinate series, together with assigned distortion budgets and empirical distortion-budget deviations for the learned points. The accompanying README gives the full filenames, dependencies and commands. References [66,67,68] are cited in the Supplementary Materials.

## Abbreviations

The following abbreviations are used in this manuscript:

GMM	Gaussian mixture model
MAP	maximum a posteriori
MILP	mixed-integer linear program
MMSE	minimum mean-squared error
MSE	mean-squared error

## Appendix A. Finite-Alphabet Optimization and the Exact Binary Tradeoff Curve

All sums in this appendix range over the finite alphabets defined in Section 2.2, with prior $\pi_{x}=P\left(X=x\right)$. The quantity $P_{F|X}\left(f|x\right)$ is a release-channel probability, while $W_{Y}\left(y|x\right)$ and $W_{Z_{i}}\left(z_{i}|x\right)$ are side-information-channel probabilities.

### Appendix A.1. MAP Risks and the MILP Formulation

For a finite observation *O*, a maximum a posteriori estimator selects a source symbol with the largest joint mass P(X=x,O=o). Its correct-decision probability is the sum, over observations, of the largest joint mass across source symbols [40,41]. If several symbols maximize the joint mass, any tied choice gives the same conditional Hamming risk. Accordingly, the risk formulas below do not depend on the selector.

(A1) $$\begin{array}{cc} L_{R}\left(P_{F|X}\right) & =1-\sum_{f,y}\max_{x}\pi_{x}P_{F|X}\left(f|x\right)W_{Y}\left(y|x\right),\\ L_{A_{i}}\left(P_{F|X}\right) & =1-\sum_{f,z_{i}}\max_{x}\pi_{x}P_{F|X}\left(f|x\right)W_{Z_{i}}\left(z_{i}|x\right). \end{array}$$

To write one formulation for all three estimators, let $\left(S_{j},S_{j},W_{j}\right)$ equal $\left(Y,Y,W_{Y}\right)$ for j=R and $\left(Z_{i},Z_{i},W_{Z_{i}}\right)$ for $j=A_{i}$. Define

$$q_{f,s,x}^{j}:=\pi_{x}P_{F|X}\left(f|x\right)W_{j}\left(s|x\right).$$

The MILP represents each maximum with a nonnegative auxiliary variable and one-hot binary selectors. The one-hot selector may choose any maximizing source symbol for each observation tuple. Tied choices give the same objective value. The inequalities force the auxiliary variable to equal that selected joint mass. For every unselected alternative, the added constant relaxes its upper bound. Each joint mass lies in [0,1], so the constant one is valid [69].

The release-channel constraints are

(A2) $$\sum_{f\in F}P_{F|X}\left(f|x\right)=1,\;P_{F|X}\left(f|x\right)\geq 0.$$

For every j,f,s, impose

(A3) $$\begin{array}{cc} b_{f,s,x}^{j} & \in \left\{0,1\right\},\;m_{f,s}^{j}\geq 0,\;\sum_{x}b_{f,s,x}^{j}=1,\\ q_{f,s,x}^{j} & \leq m_{f,s}^{j}\leq q_{f,s,x}^{j}+1-b_{f,s,x}^{j},\;x\in X. \end{array}$$

Using *t* for the objective value, the reconstruction distortion constraint bounds the reconstruction Hamming distortion, and the two adversarial-loss constraints bound the adversarial Hamming losses:

(A4) $$\sum_{f,y}m_{f,y}^{R}\geq 1-D,\;t\leq 1-\sum_{f,z_{i}}m_{f,z_{i}}^{A_{i}},\;i\in \left\{1,2\right\}.$$

**Proposition** **A1** (Finite-alphabet MILP equivalence)**.** *For fixed finite source, side-information, and release alphabets, consider the program below.*

(A5) $$\max_{P_{F|X},m,b,t}t\;\mathrm{subject}\;\mathrm{to}\;\left(A2\right)–\left(A4\right).$$

*The program in (A5) has value $\gamma^{⋆}\left(D\right)$.*

**Proof.** The lower bounds in (A3) make $m_{f,s}^{j}$ at least every $q_{f,s,x}^{j}$. The one-hot selector and the big-*M* upper bound with constant 1 make it no larger than the selected term. Hence $m_{f,s}^{j}$ equals the largest $q_{f,s,x}^{j}$ over *x*. Equation (A1) then converts (A4) into $L_{R}\left(P_{F|X}\right)\leq D$ and $t\leq L_{A_{i}}\left(P_{F|X}\right)$. For any feasible release channel, choose one maximizing source symbol for each observation tuple and set its selector to one. Set the corresponding auxiliary variable equal to that maximum. Then set *t* to the minimum of the two adversarial Hamming losses. These choices are feasible, so the two optimization values are equal. □

### Appendix A.2. Binary MAP Branches and Exact Tradeoff Curve

For binary source and release alphabets, write

(A6) $$P_{F|X}=\left(\begin{array}{cc} s_{0} & 1-s_{0}\\ 1-s_{1} & s_{1} \end{array}\right),\;0\leq s_{0},s_{1}\leq 1.$$

Define the posterior-mass coordinates by $u:=\pi_{0}P_{F|X}\left(0|0\right)$ and $v:=\pi_{1}P_{F|X}\left(0|1\right)$. Then $0\leq u\leq \pi_{0}$ and $0\leq v\leq \pi_{1}$. For $j\in \{R,A_{1},A_{2}\}$, use $W_{R}:=W_{Y}$ and $W_{A_{i}}:=W_{Z_{i}}$. The binary MAP error has the following posterior-mass form.

(A7) $$\begin{array}{cc} L_{j}\left(u,v\right)= & \sum_{s\in \{0,1\}}\min\;\left\{uW_{j}\left(s|0\right),vW_{j}\left(s|1\right)\right\}\\ \; & +\sum_{s\in \{0,1\}}\min\;\left\{\left(\pi_{0}-u\right)W_{j}\left(s|0\right),\left(\pi_{1}-v\right)W_{j}\left(s|1\right)\right\}. \end{array}$$

For each pair (f,s), the MAP error equals the smaller of the two source-symbol joint masses. Equation (A7) follows by summing those error masses.

A MAP branch fixes the maximizing source symbol for every reconstructor or adversary observation. The linear inequalities under which those choices remain optimal form a polyhedral region, called a MAP-consistency cell. On each such cell, the reconstruction MAP distortion and both adversarial MAP losses are affine functions of $\left(s_{0},s_{1}\right)$.

**Lemma** **A1** (Branchwise affine reduction)**.** *For fixed decisions $\left(\delta_{R},\delta_{1},\delta_{2}\right)$, the channels for which those decisions are MAP-consistent form a polyhedron in $\left(s_{0},s_{1}\right)$. On that polyhedron, the two-adversary max–min problem at fixed D is a linear program.*

**Proof.** MAP consistency requires the chosen symbol to have joint mass at least as large as every competing symbol. For the reconstructor, for example,

$$\pi_{\delta_{R}\left(f,y\right)}P_{F|X}\left(f|\delta_{R}\left(f,y\right)\right)W_{Y}\left(y|\delta_{R}\left(f,y\right)\right)\geq \pi_{x}P_{F|X}\left(f|x\right)W_{Y}\left(y|x\right)$$

for every x,f,y, with analogous inequalities for both adversaries. The channel entries in (A6) are affine in $\left(s_{0},s_{1}\right)$, so these inequalities are linear. Within a consistent cell, each maximum in (A1) is its selected affine term. □

**Proposition** **A2** (Exact tradeoff curve for the reported binary instance)**.** *For the parameters in Section 5.3, the exact curve has five affine segments. Their endpoints and coefficients are rational numbers. For use in the exact curve and its attaining channel, define the following values.*

$$d_{1}:=\frac{17}{520},\;d_{2}:=\frac{119}{2400},\;d_{3}:=\frac{969}{6400},\;d_{4}:=\frac{4257}{24800},\;d_{5}:=\frac{89}{400}.$$

*The values $d_{1},\ldots ,d_{4}$ are the positive breakpoints of the exact tradeoff curve. The value $d_{5}$ is the side-information-only reconstruction Hamming distortion and is used in the attaining channel below.*

(A8)
$$\gamma^{⋆}\left(D\right)=\left\{\begin{array}{cc} D, & 0\leq D\leq d_{1},\\ \frac{20}{7}D-\frac{17}{280}, & d_{1}\leq D\leq d_{2},\\ \frac{19}{14}D+\frac{153}{11200}, & d_{2}\leq D\leq d_{3},\\ \frac{155}{206}D+\frac{867}{8240}, & d_{3}\leq D\leq d_{4},\\ \frac{15}{64}, & D\geq d_{4}. \end{array}\right.$$

*The positive breakpoints are approximately 0.0327, 0.0496, 0.1514, and 0.1717. Different model parameters can change their number and locations.*

**Proof.** This is a computer-assisted exact proof using rational arithmetic. Let g(D) denote the right-hand side of Equation (A8). For any release channel, set $r:=L_{R}\left(u,v\right)$ and $a_{i}:=L_{A_{i}}\left(u,v\right)$. Exact evaluation on the MAP-consistency cells gives

(A9) $$\begin{array}{cccc} a_{i} & \leq r, & \; & 0\leq r\leq d_{1},\;i\in \left\{1,2\right\},\\ a_{i} & \leq \frac{20}{7}r-\frac{17}{280}, & & d_{1}\leq r\leq d_{2},\;i\in \left\{1,2\right\},\\ a_{2} & \leq \frac{19}{14}r+\frac{153}{11200}, & & d_{2}\leq r\leq d_{3},\\ \frac{109a_{1}+97a_{2}}{206} & \leq \frac{155}{206}r+\frac{867}{8240}, & & d_{3}\leq r\leq d_{4},\\ a_{1} & \leq \frac{15}{64}, & & r\geq d_{4}. \end{array}$$

Within each MAP-consistency cell and stated risk band, every residual is affine, so its maximum occurs at a vertex. The six observer-and-release-symbol MAP terms each have three affine branch choices, giving $3^{6}=729$ raw branch assignments. Exact rational checks cover 71 nonempty cells and 158 distinct parametric LP vertices. Each of the seven scalar inequalities has maximum residual zero on its relevant vertices. The accompanying finitealphabetexactbounds.py verifies these bounds. The fourth bound also upper-bounds $\min\{a_{1},a_{2}\}$ because its positive weights sum to one. The final bound follows from Adversary 1’s release-ignoring error 15/64. For each inequality, the verification intersects every MAP-consistency cell with its stated risk band. Each nonempty intersection is a compact polytope in (u,v). Its vertices can include intersections created by the risk-band endpoints. They need not coincide with the parametric LP vertices used to construct the optimal-channel path. The residual is evaluated at every vertex of this intersection. An affine residual that is nonpositive at these vertices is nonpositive throughout the intersection. The closed cells also include boundaries where MAP decisions tie. Their union covers every binary release channel. Rational arithmetic, therefore, certifies the upper bounds over the full channel class rather than only at sampled channel parameters. Consequently, $\min\left\{a_{1},a_{2}\right\}\leq g\left(r\right)$. Since *g* is nondecreasing and feasibility gives r≤D, every feasible channel satisfies $\min\left\{a_{1},a_{2}\right\}\leq g\left(D\right)$. An attaining posterior-mass pair is

(A10) $$\begin{array}{cccc} u(D)\; & =\left\{\begin{array}{cc} \frac{23}{40}-D,\; & 0\leq D\leq d_{1},\\ \frac{89}{140}-\frac{20}{7}D,\; & d_{1}\leq D\leq d_{3},\\ -\frac{11}{4120}+\frac{140}{103}D,\; & d_{3}\leq D\leq d_{4},\\ \frac{979}{970}-\frac{440}{97}D,\; & d_{4}\leq D\leq d_{5},\\ 0,\; & D\geq d_{5}, \end{array}\right.\; & \;v(D)\; & =\left\{\begin{array}{cc} 0,\; & 0\leq D\leq d_{3},\\ -\frac{969}{4120}+\frac{160}{103}D,\; & d_{3}\leq D\leq d_{4},\\ \frac{267}{1940}-\frac{60}{97}D,\; & d_{4}\leq D\leq d_{5},\\ 0,\; & D\geq d_{5}. \end{array}\right. \end{array}$$

Every displayed pair lies in [0,23/40]×[0,17/40]. It defines a release channel through $P_{F|X}\left(0|0\right)=\frac{40u(D)}{23},\;P_{F|X}\left(0|1\right)=\frac{40v(D)}{17},$ with complementary probabilities for F=1. For $D\leq d_{5}$, substitution gives $L_{R}=D$ and $\min\left\{a_{1},a_{2}\right\}=g\left(D\right)$. For $D\geq d_{5}$, the pair (0,0) gives $L_{R}=d_{5}\leq D$ and $\min\{a_{1},a_{2}\}=15/64$. This proves Equation (A8). □

The files binaryverification.json and binaryparametricvertices.csv record the exact checks and the 158 parametric LP vertices with their budget intervals and active constraints. Table A1 uses separately recomputed risk-band intersections. Since the reconstructor can ignore the release, every channel satisfies $r\leq d_{5}$.

**Table A1:** Exact residual checks for the seven scalar upper bounds in Equation (A9). The notation *r*, $a_{i}$ and $d_{j}$ is defined above. Each row marked i=1,2 records two separate checks. Each maximum is taken over all MAP-consistency cells intersected with the indicated risk band.

Risk Band	Residual	Exact Maximum
$\left[0,d_{1}\right]$	$a_{i}-r\;\left(i=1,2\right)$	0
$\left[d_{1},d_{2}\right]$	$a_{i}-\frac{20}{7}r+\frac{17}{280}\;\left(i=1,2\right)$	0
$\left[d_{2},d_{3}\right]$	$a_{2}-\frac{19}{14}r-\frac{153}{11200}$	0
$\left[d_{3},d_{4}\right]$	$\frac{109a_{1}+97a_{2}}{206}-\frac{155}{206}r-\frac{867}{8240}$	0
$\left[d_{4},d_{5}\right]$	$a_{1}-\frac{15}{64}$	0

Along this witness, the adversarial Hamming losses coincide on $\left[0,d_{2}\right]$ and $\left[d_{3},d_{4}\right]$. Adversary 2 has the smaller Hamming loss for $d_{2}<D<d_{3}$, while Adversary 1 does so for $D>d_{4}$. Exchanging the public release labels yields an equivalent channel.

At D=1/10 every optimal binary channel has $L_{A_{1}}-L_{A_{2}}=47/800$. At D=9/50 the optimal differences range from −207/15520 to zero. Maximizing $L_{A_{1}}+L_{A_{2}}$ among the primary optimizers selects the negative endpoint. The exact verification program checks these optimal-face extrema.

### Appendix A.3. Symmetric M-Ary Specialization

Assume a uniform prior, symmetric *M*-ary side-information channels with crossover probabilities $q_{Y},q_{Z_{1}},q_{Z_{2}}$, and a symmetric release channel with $q_{F}\in \left[0,\left(M-1\right)/M\right]$. A crossover-*q* channel outputs the source symbol with probability 1−q and each of the other M−1 symbols with probability q/(M−1). The resulting reconstruction MAP distortion and adversarial MAP losses are [40,41]

(A11) $$L_{R}=\min\left\{q_{Y},q_{F}\right\},\;L_{A_{i}}=\min\left\{q_{Z_{i}},q_{F}\right\}.$$

For M=2, the corresponding derivation of the reconstruction MAP distortion and adversarial MAP losses appears in ([70], Appendix B). With $q_{\min}:=\min\left\{q_{Z_{1}},q_{Z_{2}}\right\}$, optimizing the reconstruction MAP distortion and adversarial MAP losses in (A11) over $q_{F}$ gives the tradeoff below.

(A12) $$\underset{\begin{array}{c} 0\leq q_{F}\leq \left(M-1\right)/M\\ L_{R}\left(q_{F}\right)\leq D \end{array}}{\sup}\min_{i\in \{1,2\}}L_{A_{i}}\left(q_{F}\right)=\left\{\begin{array}{cc} \min\{D,q_{\min}\}, & D<q_{Y},\\ q_{\min}, & D\geq q_{Y}. \end{array}\right.$$

Equation (A12) is achieved by choosing $q_{F}=D$ for $D<q_{Y}$ and $q_{F}=\left(M-1\right)/M$ for $D\geq q_{Y}$. The same $q_{\min}$ always caps the objective, so this symmetric family preserves a fixed adversarial ordering or tie across all distortion budgets.

## Appendix B. Gaussian and Gaussian-Mixture Posterior Formulas

This appendix gives the formulas used for the scalar-Gaussian result, the Gaussian-mixture reference curves, and the fitted-GMM reference curves reused with MNIST [38,39,42,71,72].

### Appendix B.1. Proof of the Scalar-Gaussian Tradeoff Proposition

**Proof of Proposition** **1.** A derivation of the scalar-Gaussian result appears in ([70], Appendix A). For side information *S*, let $V_{S}:=\mathrm{Var}\left(X\;|\;S\right)$. Scalar-Gaussian conditioning gives [38,39]

(A13) $$\mathrm{Var}\left(X\;|\;F,S\right)=\frac{V_{S}}{1+UV_{S}},\;U=\frac{u^{2}}{v^{2}}.$$

For each adversary, conditional variance decreases with effective release SNR. Hence the smallest feasible SNR maximizes the minimum adversarial conditional variance. The smallest feasible *U* is 0 when $D\geq V_{Y}$, $D^{-1}-V_{Y}^{-1}$ when $0<D<V_{Y}$, and +∞ when D=0. Substitution into (A13) gives $\mathrm{Var}\left(X\;|\;F,Z_{i}\right)=\frac{D^{\prime }}{1+Q_{i}D^{\prime }},\;D^{\prime }=\min\left\{D,V_{Y}\right\},$ at that smallest feasible SNR. For $0\leq t\leq V_{Y}$ the denominator satisfies

$$1+Q_{i}t=\left(1-\frac{t}{V_{Y}}\right)+\frac{t}{V_{Y}}\frac{V_{Y}}{V_{Z_{i}}}\geq \min\;\left\{1,\frac{V_{Y}}{V_{Z_{i}}}\right\}>0.$$

Hence $g_{i}^{\prime }\left(t\right)={\left(1+Q_{i}t\right)}^{-2}>0$ throughout the interval. Taking the minimum over *i* proves (3). For jointly Gaussian variables, conditional means are affine, so MMSE and linear MMSE coincide within this release family [38,39]. Finally, $g_{i}\left(t\right):=t/\left(1+Q_{i}t\right)$ is increasing on $\left[0,V_{Y}\right]$ and satisfies $g_{i}\left(V_{Y}\right)=V_{Z_{i}}$, hence $g_{i}\left(D^{\prime }\right)\leq V_{Z_{i}}$. □

### Appendix B.2. Gaussian-Mixture Posterior

A component label is said to be unobserved when its value is unavailable to the estimators. Let the unobserved label $C\in \{1,\ldots ,M_{C}\}$ have probability $\pi_{c}$, and let $X\in R^{n_{x}}$. The component-conditional source distribution is given by $X|C=c∼N(\mu_{c},\Sigma_{c})$. Assume $\Sigma_{c}≻0$ for every component. Write the linear release with additive Gaussian noise as $F=A_{F}X+N_{F}$, where $N_{F}∼N\left(0,\Sigma_{F}\right)$. For observer *j*, let $S_{j}=H_{j}X+N_{j}$, where j=R denotes the reconstructor and $j=A_{i}$ denotes adversary *i*. Let $\Sigma_{j}$ denote the covariance of $N_{j}$, and assume all channel noises are mutually independent and independent of (X,C). Assume $\Sigma_{F}≻0$ and $\Sigma_{j}≻0$ for every observer *j*. The stacked release and side information are defined below.

$$O_{j}:=\left[\begin{array}{c} F\\ S_{j} \end{array}\right]=B_{j}X+N_{O,j},\;B_{j}:=\left[\begin{array}{c} A_{F}\\ H_{j} \end{array}\right],\;\Lambda_{j}:=\mathrm{diag}\left(\Sigma_{F},\Sigma_{j}\right).$$

Here, $N_{O,j}∼N\left(0,\Lambda_{j}\right)$. Conditional on C=c, the following expressions give the posterior covariance and mean of X.

(A14) $$\Sigma_{X|j,c}={\left(\Sigma_{c}^{-1}+B_{j}^{T}\Lambda_{j}^{-1}B_{j}\right)}^{-1},\;\mu_{X|j,c}\left(o\right)=\Sigma_{X|j,c}\left(\Sigma_{c}^{-1}\mu_{c}+B_{j}^{T}\Lambda_{j}^{-1}o\right).$$

Let ϕ(·;m,Γ) denote the Gaussian density with mean m and covariance Γ. The following expression gives the posterior probability of component *c*.

$$\omega_{c,j}\left(o\right)=\frac{\pi_{c}\phi \left(o;B_{j}\mu_{c},B_{j}\Sigma_{c}B_{j}^{T}+\Lambda_{j}\right)}{\sum_{\ell =1}^{M_{C}}\pi_{\ell }\phi \left(o;B_{j}\mu_{\ell },B_{j}\Sigma_{\ell }B_{j}^{T}+\Lambda_{j}\right)}.$$

Define ${\bar{\mu }}_{j}\left(o\right):=\sum_{c}\omega_{c,j}\left(o\right)\mu_{X|j,c}\left(o\right)$. The following expression gives the mixture posterior covariance.

(A15) $$\mathrm{Var}\left(X\;|\;O_{j}=o\right)=\sum_{c}\omega_{c,j}\left(o\right)\left(\Sigma_{X|j,c}+\mu_{X|j,c}\left(o\right)\mu_{X|j,c}{\left(o\right)}^{T}\right)-{\bar{\mu }}_{j}\left(o\right){\bar{\mu }}_{j}{\left(o\right)}^{T}.$$

In (A15), $\sum_{c}\omega_{c,j}\left(o\right)\Sigma_{X|j,c}$ averages within-component posterior covariance. The remaining posterior-mean terms measure dispersion among component-specific posterior means because *C* remains unobserved. For the isotropic release, set $A_{F}=\alpha_{G}I_{n_{x}}$ and $\Sigma_{F}=\sigma_{F}^{2}I_{n_{x}}$, and define $U=\alpha_{G}^{2}/\sigma_{F}^{2}$.

$$\Sigma_{X|j,c}\left(U\right)={\left(\Sigma_{c}^{-1}+UI_{n_{x}}+H_{j}^{T}\Sigma_{j}^{-1}H_{j}\right)}^{-1}.$$

The expectation $L_{j}\left(U\right)=E\left[n_{x}^{-1}\mathrm{tr}\mathrm{Var}\left(X\;|\;F,S_{j}\right)\right]$ defines model-based reconstruction distortion for j=R and adversarial estimation loss for $j=A_{i}$. For each realized observation, Equations (A14) and (A15) give the posterior quantities analytically. The expectation defining $L_{j}\left(U\right)$ is approximated by Monte Carlo because the posterior weights depend on the random observation.

### Appendix B.3. Monotonicity in the Effective Release SNR

**Proof of Lemma** **1.** Write $G_{U}=\sqrt{U}\;X+\varepsilon$. Here $\varepsilon ∼N(0,I_{n_{x}})$ is independent of $\left(X,S_{j}\right)$. For U>0 this observation is an invertible rescaling of the normalized release. For almost every s define

$$m_{j}\left(U,s\right):=\frac{1}{n_{x}}E\;\left[{∥X-E[X|G_{U},S_{j}=s]∥}^{2}\;|\;S_{j}=s\right].$$

Then $L_{j}\left(U\right)=\int m_{j}\left(U,s\right)\;P_{S_{j}}\left(ds\right)$ and $0\leq m_{j}\left(U,s\right)\leq \frac{1}{n_{x}}{E[∥X∥}^{2}\left|S_{j}=s\right]$. The upper bound is integrable. Conditioned on $S_{j}=s$, the source remains a finite Gaussian mixture. Its component weights, means and positive-definite covariances are $p_{c}\left(s\right)$, $m_{c}\left(s\right)$ and $V_{c}$, respectively. For fixed s the posterior component weights after observing $G_{U}=g$ are proportional to $p_{c}\left(s\right)\;\phi \left(g;\sqrt{U}\;m_{c}\left(s\right),I_{n_{x}}+UV_{c}\right)$. The corresponding component means are

$$m_{c}\left(s\right)+\sqrt{U}\;V_{c}{\left(I_{n_{x}}+UV_{c}\right)}^{-1}\left(g-\sqrt{U}\;m_{c}\left(s\right)\right).$$

Every expression is continuous for finite U≥0. At U=0 the posterior weights equal $p_{c}\left(s\right)$ and the posterior mean equals $E[X|S_{j}=s]$. Fix s and a finite B>0. Set $M_{s}=\max_{c}∥m_{c}\left(s\right)∥$ and $V_{\max}=\max_{c}{∥V_{c}∥}_{\mathrm{op}}$. For 0≤U≤B the full posterior mean has norm at most

$$M_{s}+\sqrt{B}\;V_{\max}\left(∥G_{U}∥+\sqrt{B}\;M_{s}\right),\;∥G_{U}∥\leq \sqrt{B}\;∥X∥+∥\varepsilon ∥.$$

These bounds provide a square-integrable envelope under the conditional law of (X,ε). Dominated convergence makes $m_{j}\left(U,s\right)$ continuous on [0,B], including at zero. The integrable bound above then makes $L_{j}\left(U\right)$ continuous at every finite SNR. In particular $L_{j}\left(0\right)=\frac{1}{n_{x}}E[∥X-E\left[X|S_{j}\right]{∥}^{2}]$. For U>0 the estimator $G_{U}/\sqrt{U}$ has per-coordinate MSE 1/U. Hence $0\leq L_{j}\left(U\right)\leq 1/U$. This proves continuity at the endpoint $L_{j}\left(+\infty \right)=0$. For $0<U_{1}<U_{2}<+\infty$ define ${\tilde{F}}_{U}=X+U^{-1/2}\varepsilon$. Then

(A16) $$\left(X,S_{j},{\tilde{F}}_{U_{1}}\right)\overset{d}{=}\left(X,S_{j},{\tilde{F}}_{U_{2}}+N_{\mathrm{add}}\right),$$

where $N_{\mathrm{add}}∼N\;\left(0,\left(U_{1}^{-1}-U_{2}^{-1}\right)I_{n_{x}}\right)$ is independent of $\left(X,S_{j},{\tilde{F}}_{U_{2}}\right)$. An estimator with release precision $U_{2}$ can generate a release with the distribution at $U_{1}$. Bayes risk, therefore, cannot increase with *U* [65]. Continuity extends this ordering to both endpoints. Finally U↦U/(1+U) identifies [0,∞] with the compact interval [0,1]. The nonempty feasible set is closed. Its smallest element $U_{D}$, therefore, exists. □

### Appendix B.4. MNIST Effective Observation

For the MNIST assessment against the fitted-GMM reference curves, $F=X+N_{F}$ and $S_{j}=X+N_{j}$ have independent isotropic Gaussian noises. Their pair contains the same estimation information as the precision-weighted observation below [38,39].

(A17) $$\sigma_{\mathrm{eff},j}^{2}\left(\frac{F}{\sigma_{F}^{2}}+\frac{S_{j}}{\sigma_{j}^{2}}\right)=X+N_{\mathrm{eff},j},\;\sigma_{\mathrm{eff},j}^{2}=\frac{\sigma_{F}^{2}\sigma_{j}^{2}}{\sigma_{F}^{2}+\sigma_{j}^{2}}.$$

In (A17), $N_{\mathrm{eff},j}∼N\left(0,\sigma_{\mathrm{eff},j}^{2}I_{n_{x}}\right)$ and is independent of (X,C). The mixture formulas can, therefore, be applied directly to this sufficient observation. Digit labels are used to estimate the component parameters but are unobserved during posterior evaluation.

## Appendix C. Proof of the Single-Adversary Finite-Iteration Bounds

The proof uses conditional expectations and a step-size-weighted squared distance. Related stochastic-approximation recursions appear in [48,49,73].

**Proof of Theorem** **1.** Let $A_{t}=1_{E_{t}}$ and $M_{t}=E\left[A_{t}V_{t}\right]$. Expressions multiplied by $A_{t}$ are defined as zero outside $E_{t}$. We prove $M_{t}<\infty$ by induction, starting from $M_{0}=V_{0}$. Fix t∈{0,…,T−1} with $M_{t}<\infty$ and define

$$e_{t}^{p}:=p_{t}-p^{⋆},\;e_{t}^{r}:=r_{t}-r^{⋆},\;e_{t}^{a}:=a_{t}-a^{⋆}.$$

Set $h_{t}^{j}:=G_{j}\left(\theta_{t}\right)$ on $E_{t}$ and $h_{t}^{j}:=0$ otherwise for j∈{p,r,a}. Since $A_{t}$ is $F_{t}$-measurable, it remains explicit in each conditional expectation. The local Lipschitz bound gives

$$A_{t}{∥G\left(\theta_{t}\right)∥}^{2}\leq L^{2}A_{t}{∥\theta_{t}-\theta^{⋆}∥}^{2}\leq L^{2}\max\left\{\alpha ,\beta \right\}A_{t}V_{t}.$$

Thus the restricted current operator is square integrable. Assumption 1 makes $A_{t}g_{t}^{p}$ square integrable and gives the conditional second-moment identity

$$\begin{array}{cc} E[A_{t}{∥g_{t}^{p}∥}^{2}|F_{t}] & =A_{t}{∥h_{t}^{p}∥}^{2}+E\left[A_{t}{∥g_{t}^{p}-h_{t}^{p}∥}^{2}|F_{t}\right]\\ & \leq A_{t}\left({∥h_{t}^{p}∥}^{2}+\sigma^{2}\right). \end{array}$$

Expanding the privatizer update yields

(A18) $$\begin{array}{c} E\;\left[A_{t}\alpha^{-1}{∥p_{t+1}-p^{⋆}∥}^{2}|F_{t}\right]\leq A_{t}\left(\alpha^{-1}{∥e_{t}^{p}∥}^{2}-2\langle e_{t}^{p},h_{t}^{p}\rangle +\alpha {∥h_{t}^{p}∥}^{2}+\alpha \sigma^{2}\right). \end{array}$$

For b∈{r,a}, introduce comparison gradients used only in the proof. Define

$$\left({\bar{g}}_{t}^{b},\delta_{t}^{b}\right):=\left\{\begin{array}{cc} \left(g_{t}^{b},G_{b}\left(\theta_{t+1/2}\right)-h_{t}^{b}\right), & E_{t}\;\mathrm{and}\;\theta_{t+1/2}\in U,\\ (h_{t}^{b},0), & \mathrm{otherwise}, \end{array}\right.$$

and set ${\tilde{h}}_{t}^{b}:=h_{t}^{b}+\delta_{t}^{b}$ and ${\bar{b}}_{t+1}:=b_{t}-\beta {\bar{g}}_{t}^{b}$. Let ${\bar{V}}_{t+1}$ use the actual $p_{t+1}$ and these comparison estimator states. The branch choice and $A_{t}$ are $F_{t+1/2}$-measurable. On the first branch both operator evaluation states lie in U, while $\delta_{t}^{b}=0$ on the second. The identity $\theta_{t+1/2}-\theta_{t}=\left(-\alpha g_{t}^{p},0,0\right)$ therefore gives $A_{t}∥\delta_{t}^{b}∥\leq L\alpha A_{t}∥g_{t}^{p}∥$. Hence $A_{t}\delta_{t}^{b}$ and $A_{t}{\tilde{h}}_{t}^{b}$ are square integrable. Since $A_{t}\left({\bar{g}}_{t}^{b}-{\tilde{h}}_{t}^{b}\right)=A_{t}\xi_{t}^{b}$, Assumption 1 also makes $A_{t}{\bar{g}}_{t}^{b}$ square integrable and gives

$$\begin{array}{c} E\left[A_{t}{\bar{g}}_{t}^{b}|F_{t+1/2}\right]=A_{t}{\tilde{h}}_{t}^{b},\\ E\;\left[A_{t}{∥{\bar{g}}_{t}^{b}-{\tilde{h}}_{t}^{b}∥}^{2}|F_{t+1/2}\right]\leq A_{t}\sigma^{2}. \end{array}$$

These facts justify the squared-error expansions and tower property below. First condition on $F_{t+1/2}$ to obtain

(A19) $$\begin{array}{c} E\;\left[A_{t}\beta^{-1}{∥{\bar{b}}_{t+1}-b^{⋆}∥}^{2}|F_{t+1/2}\right]\leq A_{t}\left(\beta^{-1}{∥e_{t}^{b}∥}^{2}-2\langle e_{t}^{b},{\tilde{h}}_{t}^{b}\rangle +\beta {∥{\tilde{h}}_{t}^{b}∥}^{2}+\beta \sigma^{2}\right). \end{array}$$

Applying the tower property to (A19) gives

(A20) $$\begin{array}{cc} E\;\left[A_{t}\beta^{-1}{∥{\bar{b}}_{t+1}-b^{⋆}∥}^{2}|F_{t}\right]\; & \leq A_{t}\beta^{-1}{∥e_{t}^{b}∥}^{2}-2E\left[A_{t}\langle e_{t}^{b},{\tilde{h}}_{t}^{b}\rangle |F_{t}\right]\\ \; & \;+\beta E\left[A_{t}{∥{\tilde{h}}_{t}^{b}∥}^{2}|F_{t}\right]+A_{t}\beta \sigma^{2}. \end{array}$$

The shift estimate and the privatizer second-moment bound imply

(A21) $$\begin{array}{c} A_{t}∥\delta_{t}^{b}∥\leq L\alpha A_{t}∥g_{t}^{p}∥,\\ E\left[A_{t}{∥\delta_{t}^{b}∥}^{2}|F_{t}\right]\leq A_{t}L^{2}\alpha^{2}\left({∥h_{t}^{p}∥}^{2}+\sigma^{2}\right). \end{array}$$

Young’s inequality and (A21) imply

(A22) $$\begin{array}{c} E\left[A_{t}\langle e_{t}^{b},{\tilde{h}}_{t}^{b}\rangle |F_{t}\right]\geq A_{t}\left(\langle e_{t}^{b},h_{t}^{b}\rangle -\frac{\mu }{8}{∥e_{t}^{b}∥}^{2}-\frac{2L^{2}\alpha^{2}}{\mu }\left({∥h_{t}^{p}∥}^{2}+\sigma^{2}\right)\right), \end{array}$$

while ${∥u+v∥}^{2}\leq 2{∥u∥}^{2}+2{∥v∥}^{2}$ gives

(A23) $$E\left[A_{t}{∥{\tilde{h}}_{t}^{b}∥}^{2}|F_{t}\right]\leq A_{t}\left(2{∥h_{t}^{b}∥}^{2}+2L^{2}\alpha^{2}\left({∥h_{t}^{p}∥}^{2}+\sigma^{2}\right)\right).$$

Substituting (A22) and (A23) into (A20) gives

$$\begin{array}{cc} E\;\left[A_{t}\beta^{-1}{∥{\bar{b}}_{t+1}-b^{⋆}∥}^{2}|F_{t}\right]\; & \leq A_{t}(\beta^{-1}{∥e_{t}^{b}∥}^{2}-2\langle e_{t}^{b},h_{t}^{b}\rangle +\frac{\mu }{4}{∥e_{t}^{b}∥}^{2}+2\beta {∥h_{t}^{b}∥}^{2}\\ \; & \;+2L^{2}\alpha^{2}\left(\beta +\frac{2}{\mu }\right)\left({∥h_{t}^{p}∥}^{2}+\sigma^{2}\right)+\beta \sigma^{2}). \end{array}$$

After summing both estimator bounds, the coefficient of $A_{t}{∥h_{t}^{p}∥}^{2}$ is $\alpha +4L^{2}\alpha^{2}\left(\beta +2/\mu \right)$, while those of $A_{t}{∥h_{t}^{r}∥}^{2}$ and $A_{t}{∥h_{t}^{a}∥}^{2}$ are both 2β. The total noise coefficient equals $C_{\alpha ,\beta }$, which also dominates these three gradient coefficients. Adding (A18) and the two response bounds therefore gives

$$\begin{array}{cc} E[A_{t}{\bar{V}}_{t+1}|F_{t}] & \leq A_{t}(V_{t}-2\langle \theta_{t}-\theta^{⋆},G\left(\theta_{t}\right)\rangle +\frac{\mu }{4}\left({∥e_{t}^{r}∥}^{2}+{∥e_{t}^{a}∥}^{2}\right)\\ & \;+C_{\alpha ,\beta }{∥G\left(\theta_{t}\right)∥}^{2}+C_{\alpha ,\beta }\sigma^{2}). \end{array}$$

On $E_{t}$, strong monotonicity and $G(\theta^{⋆})=0$ give $\langle \theta_{t}-\theta^{⋆},G\left(\theta_{t}\right)\rangle \geq \mu {∥\theta_{t}-\theta^{⋆}∥}^{2}$. Since ${∥e_{t}^{r}∥}^{2}+{∥e_{t}^{a}∥}^{2}\leq {∥\theta_{t}-\theta^{⋆}∥}^{2}$,

(A24) $$\begin{array}{c} E\left[A_{t}{\bar{V}}_{t+1}|F_{t}\right]\leq A_{t}\left(V_{t}-\frac{7\mu }{4}{∥\theta_{t}-\theta^{⋆}∥}^{2}+C_{\alpha ,\beta }{∥G\left(\theta_{t}\right)∥}^{2}+C_{\alpha ,\beta }\sigma^{2}\right). \end{array}$$

The comparison update agrees with the actual update on $E_{t+1}$. Otherwise $A_{t+1}V_{t+1}=0$, while ${\bar{V}}_{t+1}\geq 0$. Since $A_{t+1}\leq A_{t}$, it follows that

$$0\leq A_{t+1}V_{t+1}\leq A_{t}{\bar{V}}_{t+1}.$$

On $E_{t}$, the Lipschitz bound $∥G\left(\theta_{t}\right)∥\leq L∥\theta_{t}-\theta^{⋆}∥$ and the definition of λ first give the residual estimate

(A25) $$E\left[A_{t+1}V_{t+1}|F_{t}\right]\leq A_{t}\left(V_{t}-\lambda {∥G\left(\theta_{t}\right)∥}^{2}+C_{\alpha ,\beta }\sigma^{2}\right).$$

Strong monotonicity and Cauchy–Schwarz, together with the definition of $V_{t}$, give

(A26) $$\begin{array}{c} A_{t}∥G\left(\theta_{t}\right)∥\geq \mu A_{t}∥\theta_{t}-\theta^{⋆}∥,\\ A_{t}{∥\theta_{t}-\theta^{⋆}∥}^{2}\geq \eta A_{t}V_{t}. \end{array}$$

For contraction, applying the Lipschitz bound directly to the positive squared-operator term in (A24) preserves the larger coefficient $7\mu /4-C_{\alpha ,\beta }L^{2}=L^{2}\lambda$. Combining this with (A26) and $c=\min\{1,\eta L^{2}\lambda \}$ yields

$$\begin{array}{cc} E[A_{t+1}V_{t+1}|F_{t}] & \leq A_{t}\left(V_{t}-\left(\frac{7\mu }{4}-C_{\alpha ,\beta }L^{2}\right){∥\theta_{t}-\theta^{⋆}∥}^{2}+C_{\alpha ,\beta }\sigma^{2}\right)\\ & =A_{t}\left(V_{t}-L^{2}\lambda {∥\theta_{t}-\theta^{⋆}∥}^{2}+C_{\alpha ,\beta }\sigma^{2}\right)\\ & \leq A_{t}\left(\left(1-\eta L^{2}\lambda \right)V_{t}+C_{\alpha ,\beta }\sigma^{2}\right)\\ & \leq A_{t}\left(\left(1-c\right)V_{t}+C_{\alpha ,\beta }\sigma^{2}\right). \end{array}$$

Taking total expectations gives

$$M_{t+1}\leq \left(1-c\right)M_{t}+C_{\alpha ,\beta }\sigma^{2}\mathrm{Pr}\left(E_{t}\right)\leq \left(1-c\right)M_{t}+C_{\alpha ,\beta }\sigma^{2}<\infty .$$

This completes the induction and also makes every restricted residual moment finite by the preceding Lipschitz estimate. Unrolling the scalar affine recursion proves, for 0≤t≤T,

$$M_{t}\leq {\left(1-c\right)}^{t}V_{0}+C_{\alpha ,\beta }\sigma^{2}\sum_{k=0}^{t-1}{\left(1-c\right)}^{k}\leq {\left(1-c\right)}^{t}V_{0}+\frac{C_{\alpha ,\beta }\sigma^{2}}{c}.$$

Since $E_{T}\subseteq E_{t}$ and $\mathrm{Pr}(E_{T})\geq 1-\delta >0$,

$$E\left[V_{t}|E_{T}\right]=\frac{E[A_{T}V_{t}]}{\mathrm{Pr}(E_{T})}\leq \frac{M_{t}}{1-\delta }\leq \frac{{\left(1-c\right)}^{t}V_{0}+C_{\alpha ,\beta }\sigma^{2}/c}{1-\delta },$$

which is (16). Finally, taking total expectations in (A25), summing from 0 to s−1, and using $M_{s}\geq 0$ gives

$$\lambda \sum_{t=0}^{s-1}E\left[A_{t}{∥G\left(\theta_{t}\right)∥}^{2}\right]\leq V_{0}+sC_{\alpha ,\beta }\sigma^{2}.$$

The minimum of *s* nonnegative terms is no larger than their average. Applying this fact and $E_{T}\subseteq E_{t}$ to the residual sum yields

$$\begin{array}{cc} \min_{0\leq t<s}E\left[{∥G\left(\theta_{t}\right)∥}^{2}|E_{T}\right] & \leq \frac{1}{s}\sum_{t=0}^{s-1}E\left[{∥G\left(\theta_{t}\right)∥}^{2}|E_{T}\right]\\ & \leq \frac{\sum_{t=0}^{s-1}E\left[A_{t}{∥G\left(\theta_{t}\right)∥}^{2}\right]}{s(1-\delta )}\\ & \leq \frac{V_{0}/\left(\lambda s\right)+C_{\alpha ,\beta }\sigma^{2}/\lambda }{1-\delta }, \end{array}$$

which proves (17). Every oracle identity used the current history, without conditioning on the future event $E_{T}$. For exact gradients of the population objectives, every conditional gradient-error term is zero, so the same argument applies with $\sigma^{2}=0$. With deterministic initialization, the recursion is then deterministic. Since $\mathrm{Pr}(E_{T})>0$, containment is certain and one can take δ=0, giving the stated exact-gradient bounds. □
