# Supplementary material for: Distortion-Constrained Minimax Data Sanitization with Multiple Adversaries
Source: Entropy (Basel). 2026 Sep 25;28(10):1054. doi: 10.3390/e28101054 (PMC13703536; doi:10.3390/e28101054)
Supplement: Supplementary file 1 [file entropy-28-01054-s001.zip › Supplementary Containment Analysis.pdf]

Supplementary Containment Analysis

Distortion-Constrained Minimax Data Sanitization with Multiple Adversaries

Amirarsalan Moatazedian[1,*], Yauhen Yakimenka[1,*], Rémi A. Chou[2] and Jörg Kliewer[1]

[1] Helen and John C. Hartmann Department of Electrical and Computer Engineering, New Jersey Institute of Technology, Newark, NJ 07102, USA; jkliewer@njit.edu

[2] Department of Computer Science and Engineering, The University of Texas at Arlington, Arlington, TX 76019, USA; remi.chou@uta.edu

[*] Correspondence: am3734@njit.edu (A.M.); yauhen.yakimenka@njit.edu (Y.Y.)

Document Organization

This document gives the complete derivations for the reference calculations summarized in Section 4.2 of the article. Sections S1–S6 treat the affine ten-component GMM game. Section S7 treats the component-conditioned scalar spectral recursion. The supporting programs and data are in `Supporting_Files/Manuscript`. Their dependencies and evaluation commands are given in `Supporting_Files/README.md`. Equation and result numbers in this document use an S prefix. References to the article retain its numbering.

Scope

Two explicit calculations examine the local assumptions for the ten-component GMM. The first uses a coupled affine privatizer and separately parameterized reconstructor and adversary with hidden component labels. The second uses the fitted covariance spectra in a component-conditioned scalar update. Each calculation specifies its own states, initialization, gradient oracle, fixed steps, and containment event. The accompanying programs evaluate their analytic bounds.

Contents

S1 Affine Gaussian-Mixture Game . . . 2
S2 Local Geometry and Unbiased Gradients . . . 3
S3 Gaussian Bounds for the Privatizer Noise . . . 4
S4 Containment of the Estimator Updates . . . 6
S5 Containment of the Privatizer Updates . . . 7
S6 Interpretation of the Three Assumptions . . . 8
S7 A Scalar Reference from the Fitted Covariance Spectra . . . 9
S7.1 Geometry and Variance . . . 9
S7.2 Invariant Intervals . . . 10
S7.3 Finite-Horizon Bounds for Larger Distortion Budgets . . . 10
S7.4 Numerical Bounds and Scope . . . 10

S1 Affine Gaussian-Mixture Game

Use the source distribution in Section 2.4 with dimension $n = 784$. The reconstruction side-information noise has variance 96 per coordinate. The two adversarial variances are 200 and 72. Adversary 2 can add an independent Gaussian vector with covariance $128\boldsymbol{I}_n$ to its side information while keeping the public sanitized release. This generates the joint distribution available to Adversary 1, so the Bayes-risk ordering in Section 5.7 applies for every release channel and distortion budget. The minimum of the two Bayes adversarial losses therefore equals the loss of Adversary 2. The single adversary in the following construction corresponds to that stronger adversary. Define

$$\boldsymbol{F} = \boldsymbol{X} + \sqrt{v(p)}\boldsymbol{\varepsilon}, \qquad \boldsymbol{S}_j = \boldsymbol{X} + \sigma_j \boldsymbol{\nu}_j, \qquad \sigma_{\mathrm{R}}^2 = 96, \quad \sigma_{\mathrm{A}}^2 = 72.$$

All noise vectors are independent standard Gaussian vectors and independent of $\boldsymbol{X}$. The component label remains hidden. With $w = 0.9$ use separately parameterized estimators

$$\widehat{\boldsymbol{X}}_{\mathrm{R}} = w\boldsymbol{F} + (1-w)\boldsymbol{S}_{\mathrm{R}} + \sqrt{n/2}\, r, \qquad \widehat{\boldsymbol{X}}_{\mathrm{A}} = w\boldsymbol{F} + (1-w)\boldsymbol{S}_{\mathrm{A}} + \sqrt{n/2}\, a.$$

Here p is the privatizer parameter and $r, a \in \mathbb{R}^n$ are estimator offsets. Both estimators use the public sanitized release and their respective side information. Their estimation errors cancel $\boldsymbol{X}$ exactly. Their risks therefore apply to the fitted GMM without observing its component label.

The upper budget $D_{\mathrm{hi}} = 42.1418$ specifies the evaluated interval, rather than an exact Bayes side-information-only distortion. For $1 \le D \le D_{\mathrm{hi}}$ and $\rho = 1000$ set

$$\begin{gathered} z = (2\rho)^{-1/2}, \qquad c_v = \frac{1}{\sqrt{2\rho}\, w^2}, \qquad v(p) = v_0 + c_v p, \\ v_0 = \frac{D + 1/(2\rho) - (1-w)^2\sigma_{\mathrm{R}}^2}{w^2}. \end{gathered} \tag{S1}$$

This coordinate scale fixes the meaning of the step size. A gradient step of size α in p corresponds to size $\alpha {c_v}^2$ in v, where $c_v^2 = 1/(2\rho w^4)$. For $10^{-5} \le \alpha \le 10^{-3}$ these sizes range from approximately 7.6208×10^{-9} to 7.6208×10^{-7}. Write $Q_j(p) = w^2 v(p) + (1-w)^2\sigma_j^2$. The expected reconstruction distortion and adversarial loss are

$$L_{\mathrm{R}} = Q_{\mathrm{R}}(p) + \tfrac{1}{2}\|r\|^2, \qquad L_{\mathrm{A}} = Q_{\mathrm{A}}(p) + \tfrac{1}{2}\|a\|^2.$$

Thus the operator in Equation (14) is

$$G(p, r, a) = \left(p + \sqrt{\rho/2}\, \|r\|^2,\ r,\ a\right). \tag{S2}$$

Its zero is $\theta^\star = (0, 0, 0)$. The stationary reconstruction distortion is $D + 1/(2\rho) = D + 0.0005$ for the squared-penalty game.

Use the neighborhood and initialization

$$\mathcal{U} = \{(p, r, a) : |p| \le 1,\ \|r\|, \|a\| \le 0.02\}, \qquad |p_0| \le 0.1, \quad r_0 = a_0 = 0. \tag{S3}$$

Let $B = (0.02)^2/2 = 0.0002$ and define

$$q_- = 1 + 0.0005 - z > 0.9781393, \qquad q_+ = D_{\mathrm{hi}} + 0.0005 + z < 42.1647.$$

Throughout $\mathcal{U}$ one has $q_- \le Q_{\mathrm{R}} \le q_+$ and $Q_{\mathrm{A}} \le q_+$. Moreover $w^2 v = Q_{\mathrm{R}} - 0.96$ and $v > 0.0223$. The entire neighborhood is therefore inside the positive-variance domain. The lower limit arises from the fixed weight and neighborhood, since

$$\min_{|p| \le 1} v(p) = \frac{D - 0.9595 - 1/\sqrt{2000}}{0.81}.$$

Positivity requires $D > 0.9595 + 1/\sqrt{2000} \approx 0.98186068$. The choice $D \geq 1$ supplies the uniform variance margin in the moment and concentration bounds below. It is not a lower feasibility limit for the source model. Section S7 treats every $0 < D < 1$ through a different scalar reference recursion. The specified updates define the recursion up to and including its first state outside $\mathcal{U}$. Keep the first outside state fixed thereafter. This continuation leaves the containment event and the conditional moments in Theorem 1 unchanged.

Choose α and β independently in $[10^{-5}, 10^{-3}]$ and keep each chosen step constant. One iteration updates the privatizer followed by the reconstructor and the adversary. The ten-component GMM uses 50000 training samples over 64 epochs. At the start of each epoch the source rows are randomly permuted and processed in consecutive groups of 256. Only complete groups generate an update, and the remaining rows are left unused for that epoch. Thus each epoch contains $\lfloor 50000/256 \rfloor = 195$ iterations and the finite horizon is $T = 64 \cdot 195 = 12480$. Every iteration uses $m = 256$. The calculation below uses this batch size and horizon with the stated fixed steps and fresh channel noises.

S2 Local Geometry and Unbiased Gradients

The symmetric Jacobian is

$$\frac{J_G + J_G^{\mathsf{T}}}{2} = \begin{bmatrix} 1 & \sqrt{\rho/2}\, r^{\mathsf{T}} & 0 \\ \sqrt{\rho/2}\, r & \boldsymbol{I}_n & 0 \\ 0 & 0 & \boldsymbol{I}_n \end{bmatrix}.$$

Its smallest eigenvalue is at least $1 - \sqrt{\rho/2}\|r\|$. The norm of $J_G - \boldsymbol{I}$ is $\sqrt{2\rho}\|r\|$. Integration along line segments in $\mathcal{U}$ verifies Assumption 2 with

$$\mu = 1 - 0.02\sqrt{500} > 0.5527, \qquad L = 1 + 0.02\sqrt{2000} < 1.8945. \tag{S4}$$

The quantity $C_{\alpha,\beta}$ in Theorem 1 increases with each positive step size. Evaluation at $\alpha = \beta = 10^{-3}$ gives

$$C_{\alpha,\beta} < 0.003052 < 0.2695 < \frac{7\mu}{4L^2}.$$

Every chosen pair therefore satisfies the theorem's numerical step-size condition.

Each privatizer batch uses $m = 256$ source rows with fresh independent Gaussian noises. Condition on the preceding history and the selected row indices. The source rows then cancel from the estimation errors, so their order within an epoch and their reuse across epochs do not affect the conditional noise laws. For sample i define

$$Z_i = \ell_{\mathrm{R},i} - D, \qquad H_i = \partial_p \ell_{\mathrm{R},i}, \qquad K_i = \partial_p \ell_{\mathrm{A},i},$$

where $\ell_{j,i}$ is the per-coordinate squared error. Use the off-diagonal estimate

$$g^p = -\frac{1}{m}\sum_i K_i + \frac{2\rho}{m(m-1)}\sum_{i \neq j} Z_i H_j. \tag{S5}$$

The off-diagonal sum equals $(\sum_i Z_i)(\sum_i H_i) - \sum_i Z_i H_i$ and has linear arithmetic cost in m. The distinct-index product is a second-order U-statistic [1]. Given the preceding history and selected row indices, the fresh noise vectors for distinct samples are independent. Consequently $\mathbb{E}[Z_i H_j] = \mathbb{E}[Z_i]\mathbb{E}[H_j]$ for $i \neq j$, and the same conditioning gives $\mathbb{E}[g^p] = G_p$. After the privatizer update each estimator uses a fresh batch of Gaussian noises. Their conditional gradient means are r and a at the respective update stages. The reconstructor loss does not depend on a, and the adversarial loss

does not depend on r. At the updated privatizer parameter, changing either estimator therefore leaves the other estimator's gradient unchanged. Their sequential updates can be grouped into one estimator step.

For $b = r$ or $b = a$ write $B_b = \|b\|^2/2$. Expansion using Isserlis' Gaussian product-moment formula [2] gives

$$\begin{gathered}\mathbb{E}\ell_j = Q_j + B_b, \qquad \mathbb{E}\partial_p \ell_j = z, \\ \operatorname{Var}(\ell_j) = \frac{2Q_j^2 + 4Q_j B_b}{n}, \qquad \operatorname{Var}(\partial_p \ell_j) = \frac{1}{\rho n} + \frac{(1-w)^2\sigma_j^2 + B_b}{2\rho w^2 n v}, \\ \operatorname{Cov}(\ell_j, \partial_p \ell_j) = \frac{2z(Q_j + B_b)}{n}.\end{gathered} \tag{S6}$$

Let $s = \mathbb{E}Z_i$ and denote the variance and covariance of Z_i, H_i by V_Z, V_H, C_{ZH}. For $U_m = [m(m-1)]^{-1}\sum_{i\neq j} Z_i H_j$ one has

$$\operatorname{Var}(U_m) = \frac{z^2 V_Z + s^2 V_H + 2zsC_{ZH}}{m} + \frac{V_Z V_H + C_{ZH}^2}{m(m-1)}. \tag{S7}$$

To obtain this identity separate the centered single-sample terms from the centered pair terms. The groups are orthogonal. Distinct centered pair terms have zero covariance unless their index sets coincide. The adversary term can be dependent on U_m. The variance triangle inequality still gives

$$\operatorname{Var}(g^p) \le \left(\sqrt{\operatorname{Var}(K_i)/m} + 2\rho\sqrt{\operatorname{Var}(U_m)}\right)^2.$$

Substitute $v > 0.0223$, $Q_j \le q_+$, $B_b \le B$ and $|s| \le z + 0.0005 + B$. With $m = 256$ the resulting bounds are

$$\operatorname{Var}(g^p) < 36, \qquad \mathbb{E}\|g^r - r\|^2,\ \mathbb{E}\|g^a - a\|^2 \le \frac{2q_+}{256} < 0.330.$$

Thus Assumption 1 holds locally with $\sigma^2 = 36$.

S3 Gaussian Bounds for the Privatizer Noise

Condition throughout on the history before a privatizer batch and its selected source-row indices. Write $Z_i^c = Z_i - s$, $H_i^c = H_i - z$ and $K_i^c = K_i - z$. The centered privatizer error decomposes as

$$\begin{gathered}\xi = g^p - G_p = L_m + R_m, \qquad L_m = -\overline{K}^c + 2\rho(z\overline{Z}^c + s\overline{H}^c), \\ R_m = \frac{2\rho}{m(m-1)}\sum_{i\neq j} Z_i^c H_j^c = \frac{2\rho}{m-1}\left(m\overline{Z}^c\overline{H}^c - \frac{1}{m}\sum_i Z_i^c H_i^c\right).\end{gathered} \tag{S8}$$

Both terms are centered and orthogonal in conditional L^2. The leading term is quadratic plus linear in the Gaussian batch. The remainder contains degree-four terms and is treated separately.

For a standard Gaussian vector g and symmetric matrix A define

$$Y = g^\top A g - \operatorname{tr}(A) + b^\top g, \qquad v_Y = 2\operatorname{tr}(A^2) + \|b\|^2, \qquad c_Y = 2\|A\|.$$

Gaussian integration gives

$$\log \mathbb{E}e^{\lambda Y} = -\tfrac{1}{2}\log\det(\boldsymbol{I} - 2\lambda A) - \lambda \operatorname{tr} A + \tfrac{\lambda^2}{2} b^\top (\boldsymbol{I} - 2\lambda A)^{-1} b.$$

The logarithmic power series and the operator norm bound imply

$$\log \mathbb{E}e^{\lambda Y} \le \frac{v_Y \lambda^2}{2(1 - c_Y|\lambda|)}, \qquad \Pr\{|Y| > \sqrt{2v_Y x} + c_Y x\} \le 2e^{-x}. \tag{S9}$$

Equation (S9) follows directly from the displayed Gaussian exponential moment for a general symmetric quadratic form plus a linear term. It uses the exponential-moment strategy for quadratic-form concentration [3]. The norm bound used below is a direct instance of that reference's positive-semidefinite result. The exponential bound holds for $c_Y|\lambda| < 1$ and the tail inequality for $x > 0$. An average of m independent copies has parameters v_Y/m and c_Y/m. These statements also hold conditionally when the preceding history fixes the coefficients.

Put $Q = Q_{\mathrm{R}}(p)$. The following normalized bounds avoid combining unrelated extreme values of Q.

Quantity	Uniform upper bound
V_Z/Q^2	0.002553
$Q^2 V_H$	0.002295
Quadratic scale of Z_i^c/Q	2/784
Quadratic scale of QH_i^c	0.002420
$\lvert C_{ZH}\rvert$	0.002406

The first bound follows directly from Equation (S6) and $B_r \le B$. For the second use

$$Q^2 V_H = \frac{1}{2\rho n}\left(2Q^2 + (0.96 + B_r)\frac{Q^2}{Q - 0.96}\right).$$

After replacing B_r by B this is convex in Q. Indeed $Q^2/(Q-b) = Q + b + b^2/(Q-b)$ for $b = 0.96$. Its maximum on $[q_-, q_+]$ is attained at an endpoint. The endpoint values are below 0.00003352 and 0.00229410. The quadratic scale of H_i^c is

$$c_H = \frac{z}{n}\left(1 + \sqrt{\frac{Q}{Q - 0.96}}\right).$$

The nonlinear term of $Q + Q^{3/2}(Q-b)^{-1/2}$ has second derivative $3b^2/[4\sqrt{Q}(Q-b)^{5/2}]$. Thus Qc_H is also convex. Its endpoint values are below 0.00023276 and 0.00241911. Finally $C_{ZH} = 2z(Q+B_r)/n$ gives the covariance bound. These constants imply

$$\mathbb{E}[R_m^2] = \frac{(2\rho)^2(V_Z V_H + C_{ZH}^2)}{m(m-1)} < 0.000714, \qquad m = 256. \tag{S10}$$

The one-sample leading term has variance at most

$$\left(\sqrt{2\rho}\sqrt{V_Z} + 2\rho|s|\sqrt{V_H} + \sqrt{V_K}\right)^2 < 9123.86.$$

For this bound use

$$\begin{gathered} V_Z \le (2q_+^2 + 4q_+ B)/784, \\ V_H \le 1/(1000 \cdot 784) + (0.96 + B)/[2000 \cdot 784(q_- - 0.96)], \\ V_K \le 1/(1000 \cdot 784) + (0.72 + B)/[2000 \cdot 784(q_- - 0.96)], \\ |s| \le z + 0.0005 + B. \end{gathered}$$

The one-coordinate quadratic matrix before division by n has norm at most

$$M_* = \sqrt{2\rho}\, q_+ + 2\rho(z + 0.0005 + B)\frac{z}{2}\left(1 + \sqrt{1 + \frac{0.96}{q_- - 0.96}}\right) + \frac{z}{2}\left(1 + \sqrt{1 + \frac{0.72}{q_- - 0.96}}\right) < 1890.1.$$

The scale for the batch average is at most $2M_*/(nm)$. Equation (S9) therefore yields

$$\log \mathbb{E}[e^{\lambda L_m} \mid \mathcal{F}] \le \frac{v_m \lambda^2}{2(1 - c_m|\lambda|)}, \qquad v_{256} = 36, \quad c_{256} < 0.01884. \tag{S11}$$

Apply Equation (S9) with $x = 31$ to each normalized pair $(Z_i^c/Q, QH_i^c)$ and its two batch averages. Let E_m be the event that all these bounds hold. A union bound gives

$$\Pr(E_m^c \mid \mathcal{F}) \le (4m + 4)e^{-31} \le \delta_0, \qquad \delta_0 = 1028e^{-31} < 3.539 \times 10^{-11}.$$

Define

$$z_1 = \sqrt{2(0.002553)31} + 2(31)/784, \qquad h_1 = \sqrt{2(0.002295)31} + (0.002420)31,$$
$$z_m = \sqrt{2(0.002553)31/m} + 2(31)/(784m), \quad h_m = \sqrt{2(0.002295)31/m} + (0.002420)31/m.$$

On E_m, Equation (S8) gives

$$|R_m| \le \frac{2000}{m-1}(m z_m h_m + z_1 h_1) < 2.90, \qquad m = 256.$$

For the probability calculation define the centered term R_m° below. The actual update continues to use R_m. Conditional centering and Cauchy–Schwarz give

$$R_m^\circ = R_m \mathbf{1}_{E_m} - \mathbb{E}[R_m \mathbf{1}_{E_m} \mid \mathcal{F}],$$
$$|\mathbb{E}[R_m \mathbf{1}_{E_m} \mid \mathcal{F}]| \le \sqrt{0.000714\delta_0} < \varepsilon_0, \qquad \varepsilon_0 = 1.6 \times 10^{-7}.$$

Consequently $|R_m^\circ| < 2.90 + \varepsilon_0$ and $\mathbb{E}[(R_m^\circ)^2 \mid \mathcal{F}] \le 0.000714$. This construction does not require an exponential moment for the unbounded degree-four term.

S4 Containment of the Estimator Updates

Check every full and intermediate state in Equation (15). Let τ be the first exit from $\mathcal{U}$. Comparison sequences retain the increment causing the first exit and set later noise increments to zero. Each survival indicator is known before its corresponding batch is drawn. All comparison noises are defined on survival and extended by zero after an earlier exit. Conditional gradient identities therefore remain valid at the required update stage.

Let I_t^e indicate survival through the state immediately before an estimator update. For its reconstructor noise $\zeta_t^r = g_t^r - r_t$ define

$$\widetilde{r}_0 = 0, \qquad \widetilde{r}_{t+1} = (1 - \beta)\widetilde{r}_t - \beta I_t^e \zeta_t^r.$$

Define $\widetilde{a}_t$ analogously. A comparison sequence agrees with the corresponding block through an update causing the first exit. On survival the centered estimator gradient is conditionally Gaussian

with covariance $2Q_j\boldsymbol{I}_n/(256n)$. With $h_e = (1-\beta)^2$, repeated conditional expectation gives a directional sub-Gaussian variance parameter. The predictable survival indicator preserves the bound, and extending the geometric sum yields

$$\begin{aligned} s_t^2 &= \frac{2q_+\beta^2}{256n}\sum_{k=0}^{t-1} h_e^{t-1-k} \le s_e^2(\beta), \\ s_e^2(\beta) &= \frac{2q_+\beta^2}{256n(1-h_e)} = \frac{2q_+\beta}{256n(2-\beta)}. \end{aligned} \tag{S12}$$

The function $\beta/(2-\beta)$ increases on $(0,1)$. Thus Equation (S12) is largest at $\beta = 10^{-3}$ and is below 2.1019×10^{-7}.

The directional exponential bound implies [3, Theorem 1]

$$\Pr\left\{\|\widetilde{r}_t\|^2 > s_e^2(\beta)(n + 2\sqrt{nx} + 2x)\right\} \le e^{-x}. \tag{S13}$$

This follows by integrating against an independent standard Gaussian vector and does not require independent accumulated coordinates. The same bound holds for $\widetilde{a}_t$. Set $T = 12480$ and $x = \log(2T/10^{-5})$. The resulting norm threshold is below $0.015121 < 0.02$. For every separately chosen fixed $\beta \in [10^{-5}, 10^{-3}]$, a union bound over both blocks and all T iterations gives

$$\Pr\{\text{first exit occurs during an estimator update by } T\} \le 10^{-5}.$$

S5 Containment of the Privatizer Updates

Let I_t indicate survival before the privatizer batch and define

$$q_0 = 0, \qquad q_{t+1} = (1-\alpha)q_t - \alpha I_t \xi_t.$$

For fixed t use weights $w_{t,k} = \alpha(1-\alpha)^{t-1-k}$. Let Q_t^L and Q_t^R be the negative weighted sums of $I_k L_{256}$ and $I_k R_{256}^\circ$, with each noise term evaluated at iteration k. On the required batch events their sum differs from q_t by at most ε_0 since $\sum_k w_{t,k} \le 1$.

Repeated conditional expectation in Equation (S11) gives

$$\log \mathbb{E} e^{\lambda Q_t^L} \le \frac{V_L\lambda^2}{2(1-C_L|\lambda|)}, \qquad V_L \ge 36\sum_{k<t} w_{t,k}^2.$$

The fixed batch size and $\alpha \le 10^{-3}$ give the uniform choices

$$\begin{aligned} V_L &= \frac{36\alpha}{2-\alpha} < 0.01800901, \\ C_L &= 0.01884\alpha \le 0.00001884, \\ V_R &= \frac{0.000714\alpha}{2-\alpha} < 0.00000035718, \\ B_R &= \alpha(2.90 + \varepsilon_0) < 0.002900001. \end{aligned} \tag{S14}$$

Every displayed bound increases with α. Evaluation at $\alpha = 10^{-3}$ therefore covers all chosen fixed steps.

A centered variable bounded by b with variance at most v satisfies the following bound for $b|\lambda| < 3$.

$$\log \mathbb{E} e^{\lambda Y} \le \frac{v\lambda^2}{2(1-b|\lambda|/3)}.$$

Expand the exponential and use $\mathbb{E}|Y|^j \le vb^{j-2}$ and $j! \ge 2\,3^{j-2}$ for $j \ge 2$. Applying this conditional bound to the centered remainder gives

$$\begin{aligned} \Pr\{|Q_t^L| \ge h\} &\le 2\exp\left[-\frac{h^2}{2(V_L + C_L h)}\right], \\ \Pr\{|Q_t^R| \ge u\} &\le 2\exp\left[-\frac{u^2}{2(V_R + B_R u/3)}\right]. \end{aligned} \tag{S15}$$

These bounds include the predictable exit indicators. They require neither independent accumulated increments nor means conditioned on future survival.

Through an update causing the first privatizer exit the exact recursion gives

$$p_t = (1-\alpha)^t p_0 - \alpha\sqrt{\rho/2}\sum_{k<t}(1-\alpha)^{t-1-k}\|r_k\|^2 + q_t.$$

The deterministic contribution has absolute value at most $0.1 + \sqrt{500}(0.02)^2$. An exit therefore requires $|q_t| > b_p$, where $b_p = 0.9 - \sqrt{500}(0.02)^2 > 0.8910557$. Take $h = 0.751$ and $u = 0.14$ so that $h + u + \varepsilon_0 < b_p$. The deterministic margin satisfies $b_p - (h + u + \varepsilon_0) > 5.5 \times 10^{-5}$. The numerical output reports this margin before rounding. The union bounds over $T = 12480$ iterations are

$$\begin{gathered} \text{Leading-term failure} < 0.004000, \\ \text{Remainder failure} < 1.075 \times 10^{-27}, \\ \text{Required batch-event failure } (T\delta_0) < 4.42 \times 10^{-7}, \\ \text{Estimator first exit} \le 10^{-5}. \end{gathered}$$

Their sum is below 0.004011. Consequently

$$\Pr\{\theta_t, \theta_{t+1/2} \in \mathcal{U}\ (0 \le t < T),\ \theta_T \in \mathcal{U}\} > 0.9959. \tag{S16}$$

For every separately fixed $D \in [1, 42.1418]$ and fixed pair $(\alpha, \beta) \in [10^{-5}, 10^{-3}]^2$, the probability that all full and intermediate states remain in $\mathcal{U}$ exceeds 99.59% over 12480 iterations of 256 samples. This statement uses $|p_0| \le 0.1$ and $r_0 = a_0 = 0$ with the fresh-noise gradient estimates above. It is a lower bound on containment probability for that fixed choice, not probability-one containment or one simultaneous event over all budgets and step pairs. The program `ten_component_gmm_containment.py` evaluates the constants from the formulas above.

The program `ten_component_gmm_containment.py` verifies the displayed numerical bounds using outward-rounded decimal intervals with 80-digit endpoints. Decimal model inputs are exact. Square roots, exponentials and logarithms are enclosed before subsequent arithmetic. The output `affine_bounds.json` records the exact endpoint strings and checked inequalities.

S6 Interpretation of the Three Assumptions

The conditional mean and variance identities verify Assumption 1 locally with $\sigma^2 = 36$. Equation (S4) verifies Assumption 2 throughout $\mathcal{U}$. Equation (S16) verifies Assumption 3 with $\delta_T(D) = 0.0041$ for each separately fixed $D \in [1, 42.1418]$. This common failure bound applies throughout the interval for the stated initialization and each separately fixed step pair. The localization argument in the proof of Theorem 1 therefore gives its conditional moment bounds for this affine process, with normalization $1/(1 - 0.0041)$. It does not require the gradient estimates to remain unbiased after conditioning on the whole containment event. The comparison used in that proof

does not alter the reference updates. Gaussian noise can cause an exit with positive probability, which is accommodated by the quantified containment assumption.

The calculation concerns the specified affine estimators and release coordinate with the off-diagonal penalty estimate in Equation (S5). The dominance of Adversary 2 explains the relevance of a single-adversary model for this source and these side-information channels. That dominance concerns Bayes losses and need not hold between losses of trained estimators. It does not identify the reference recursion with the experimental neural updates or extend the theorem to Adam with a changing learning rate. The affine estimators are not asserted to be full-mixture Bayes estimators. Conditioning on the selected rows preserves the calculation because only fresh channel noise appears in the estimation errors. The algebra also applies to a scalar Gaussian source when $n = 1$. The numerical probability above uses 784 coordinates and requires reevaluation for the scalar case.

S7 A Scalar Reference from the Fitted Covariance Spectra

This calculation connects the fitted component weights and covariance spectra to explicit scalar geometry and batch bounds. It uses component-conditioned posterior covariance. The component label defines this comparison quantity and is not released to an adversary in the source model. This quantity differs from the posterior risk with hidden component labels. The scalar calculation has one state variable and does not assert a convergence result for the three-block affine game or neural Adam training.

Let $\lambda_{c,j} > 0$ and π_c be the fitted covariance eigenvalues and component probabilities. There are ten components and 784 coordinates. For reconstruction side-noise variance 96 define

$$\begin{aligned} v_c(U) &= \frac{1}{784}\sum_{j=1}^{784} \frac{\lambda_{c,j}}{1+\lambda_{c,j}(U+1/96)}, \qquad U \geq 0, \\ L_{\text{comp}}(U) &= \sum_c \pi_c v_c(U), \qquad G_{\text{comp}}(U) = D - L_{\text{comp}}(U). \end{aligned} \tag{S17}$$

These are exact component-conditioned covariance traces for the supplied spectral model. At each iteration draw 256 fresh independent component labels with probabilities π_c and use

$$\widehat{G}_{256}(U) = D - \frac{1}{256}\sum_{i=1}^{256} v_{C_i}(U), \qquad U^+ = U - 10^{-4}\widehat{G}_{256}(U). \tag{S18}$$

This is an unbiased gradient oracle for a scalar potential with derivative G_{comp}. It averages known component risk functions rather than trained-network gradients. This independent-label oracle defines a theoretical reference. The experimental training procedure instead processes complete batches of 256 from a fresh random permutation of its training set in each epoch. The reference uses the same batch size and finite horizon without identifying these two sampling laws.

S7.1 Geometry and Variance

Differentiation gives the positive decreasing functions

$$s_c(U) = -v_c'(U) = \frac{1}{784}\sum_j \left(\frac{\lambda_{c,j}}{1+\lambda_{c,j}(U+1/96)}\right)^2.$$

On any interval $[a,b] \subset [0,\infty)$ the mean operator is strongly monotone and Lipschitz with

$$\mu = \sum_c \pi_c s_c(b), \qquad L = \sum_c \pi_c s_c(a).$$

When $0 < D < L_{\mathrm{comp}}(0)$ its stationary point is the unique solution of $L_{\mathrm{comp}}(U^*) = D$. Set $W = \max_c v_c(a) - \min_c v_c(b)$. The conditional oracle variance is at most $W^2/(4 \cdot 256)$. Fresh labels preserve this bound at adaptively chosen iterates. These formulas give explicit values of μ, L, and the variance bound from the fitted spectra and interval endpoints. The fitted inputs satisfy $\max_c s_c(0) < 3887$. Since $10^{-4} \cdot 3887 < 1$ every sampled update map is nondecreasing on $U \geq 0$.

S7.2 Invariant Intervals

The fitted inputs give $\min_c v_c(0) > 27.9449$. For $0 < D < \min_c v_c(0)$ each equation $v_c(u_c) = D$ has a positive root. Set $a = \min_c u_c$ and $b = \max_c u_c$. For any batch its update map is

$$H(U) = U + \frac{10^{-4}}{256} \sum_i (v_{C_i}(U) - D).$$

It is nondecreasing and satisfies $H(a) \geq a$ and $H(b) \leq b$. Hence $H([a, b]) \subseteq [a, b]$. Starting within this interval gives containment for every batch sequence and every horizon. Thus containment is almost sure with batch size 256. The program verifies these endpoint inequalities with directed rounding for the outward-rounded intervals in its output. In particular, every $0 < D < 1$ is included in this scalar invariance result. At $D = 0$, exact release corresponds to $U = +\infty$, outside the finite interval and finite stationary point used here.

S7.3 Finite-Horizon Bounds for Larger Distortion Budgets

For $\min_c v_c(0) < D < L_{\mathrm{comp}}(0)$ set $a = 0$ and choose b with $\max_c v_c(b) = D$. Initialize $U_0 \in [0, b]$. The upper endpoint condition holds for every batch. The lower endpoint condition holds whenever

$$\frac{1}{256} \sum_i v_{C_i}(0) \geq D.$$

Monotonicity then ensures that the entire interval maps into itself. Write $\bar{v}_0 = L_{\mathrm{comp}}(0)$ and $W_0 = \max_c v_c(0) - \min_c v_c(0)$. Hoeffding's inequality [4] and a union bound yield

$$\Pr\{\text{exit from } [0, b] \text{ by } T\} \leq \min\left\{1,\ T \exp\left[-\frac{512(\bar{v}_0 - D)^2}{W_0^2}\right]\right\}. \tag{S19}$$

This permits adaptive iterates because each successful endpoint event controls the whole update map. Fresh labels give the same endpoint probability at every iteration. The fitted values are $\bar{v}_0 \approx 42.1628$ and $W_0 \approx 20.6247$. The 99% threshold is

$$D_{99}(T) = \bar{v}_0 - W_0 \sqrt{\frac{\log(T/0.01)}{512}}.$$

With 256 samples per update and $T = 64\lfloor 50000/256 \rfloor = 12480$, directed arithmetic gives $D_{99}(12480) \geq 38.7477$. The equality case $D = \min_c v_c(0)$ also has an invariant interval with lower endpoint zero.

S7.4 Numerical Bounds and Scope

For each fixed distortion budget D, let I_D be its interval constructed above and initialize $U_0 \in I_D$. With step 10^{-4} and 256 fresh independent component labels per update, define

$$E_D = \{U_t \in I_D \text{ for every } t = 0, \ldots, 12480\}.$$

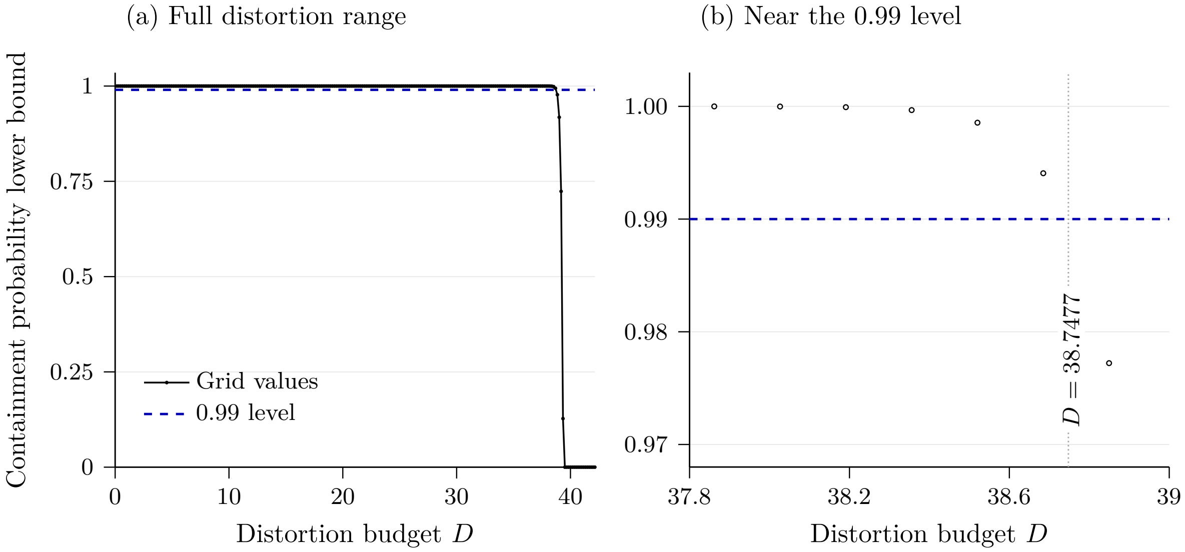

Figure S1: Lower bound on $\Pr(E_D)$ for the component-conditioned scalar spectral recursion. The event E_D means that every state remains in its specified interval over 12480 updates with step 10^{-4} and 256 fresh independent component labels per update. The plotted budgets are $D_j = 42.1418j/256$ for $j = 1, \ldots, 256$. Equation (S20) gives the numerical ranges with containment probability 1 or at least 0.99. At larger budgets, Equation (S19) gives the endpoint Hoeffding and union bound. The curve bounds containment probability for this scalar reference. It does not estimate that probability or give a convergence probability for the neural Adam recursion. A zero lower bound is uninformative. The right panel enlarges the region near the 0.99 level. Markers give the certified grid values, and connecting lines only guide the eye.

The probability that all 12481 states remain in this interval over the 12480 updates satisfies

$$\begin{aligned} \Pr(E_D) &= 1 \qquad \text{for } 0 < D \leq 27.9449, \\ \Pr(E_D) &\geq 0.99 \quad \text{for } 27.9449 < D \leq 38.7477. \end{aligned} \tag{S20}$$

Thus containment is certain in the first range and has probability at least 99% in the second range. For $38.7477 < D < \bar{v}_0$, Equation (S19) gives

$$\Pr(E_D) \geq \max\left\{0, 1 - 12480 \exp\left[-\frac{512(\bar{v}_0 - D)^2}{W_0^2}\right]\right\}.$$

This lower bound may be zero without implying failure. Each statement applies to a separately fixed D and does not assert simultaneous containment over a sweep of distortion budgets. Figure S1 evaluates the probability lower bound at 256 equally spaced positive budgets up to 42.1418. The analytic bound also applies above that plotting limit while $D < \bar{v}_0$. This endpoint belongs to the component-conditioned reference. For the hidden-label model, conditional variance also includes uncertainty in the component-conditioned posterior means, so its Bayes side-information-only risk need not equal $\bar{v}_0$.

The dependence on distortion comes from the lower endpoint of the interval. At $U = 0$, its sampled update is $10^{-4}(256^{-1} \sum_i v_{C_i}(0) - D)$. Its expected inward margin is $10^{-4}(\bar{v}_0 - D)$. As D approaches $\bar{v}_0$, this margin shrinks while finite-batch variation remains. Consequently, the concentration certificate weakens. An adverse boundary batch need not cause an actual trajectory to exit when the current state is away from that boundary. Thus a weak certificate does not establish

nonconvergence, and it gives no probability claim for the experimental neural Adam recursion. The local constants μ and L and the gradient-variance bound follow from the formulas in Section S7.1 at the corresponding interval endpoints.

The low-positive-distortion region has bound one because every possible batch preserves its interval. The endpoint $D = 0$ is excluded because its scalar stationary point requires $U \to \infty$. For finite positive spectra, writing each summand of $v_c(U)$ as $(U+\lambda_{c,j}^{-1}+1/96)^{-1}$ gives $v_c(U) \sim U^{-1}$ and $s_c(U) \sim U^{-2}$ as $U \to \infty$. Thus the component roots satisfy $u_c(D) \sim D^{-1}$ and the invariant-interval constant satisfies $\mu(D) \sim D^2$ as $D \downarrow 0$. The derivative of the mean update lies between $1 - 10^{-4}L$ and $1 - 10^{-4}\mu$. Its contraction-factor bound therefore approaches one at low distortion. This weakens the fixed-step rate guarantee even though containment remains certain. At larger budgets a bound below 0.99 gives a weaker guarantee, and a zero bound is uninformative. Neither value establishes failure of containment or determines the difficulty of neural training. For this privacy–utility problem, releasing X gives zero reconstruction distortion. A constant release gives the side-information-only risks. Interior budgets describe the design tradeoff between these directly specified endpoint mechanisms.

The program `ten_component_gmm_spectral_bounds.py` evaluates the interval endpoints, local constants, and probability bounds using the fitted weight and spectrum files. Its directed decimal arithmetic uses 60-digit precision and treats the supplied binary floating-point spectral entries as the definition of the spectral model. The resulting bounds concern that model and do not include errors from estimating its covariance matrices or computing their eigenvalues. The optional `--check-eigenvalues` command compares the spectra with the covariance matrices in `mnist_ten_component_gmm_parameters.npz`. It checks symmetry and numerical positive definiteness, then requires $|\widehat{\lambda} - \lambda| \leq 10^{-8} + 10^{-8}|\lambda|$ for every sorted eigenvalue pair. The check uses ordinary floating-point arithmetic and tests consistency of the supplied files. It is not a rigorous enclosure of eigenvalue errors. The supplied matrices pass this comparison. The program `ten_component_gmm_containment_probability.py` evaluates the bound for 12480 iterations with batch size 256 and reproduces the probability curve in Figure S1.

References

[1] Wassily Hoeffding. A class of statistics with asymptotically normal distribution. *Ann. Math. Stat.*, 19(3):293–325, 1948. https://doi.org/10.1214/aoms/1177730196.

[2] L. Isserlis. On a formula for the product-moment coefficient of any order of a normal frequency distribution in any number of variables. *Biometrika*, 12(1–2):134–139, 1918. https://doi.org/10.1093/biomet/12.1-2.134.

[3] Daniel Hsu, Sham M. Kakade, and Tong Zhang. A tail inequality for quadratic forms of subgaussian random vectors. *Electron. Commun. Probab.*, 17(52):1–6, 2012. https://doi.org/10.1214/ECP.v17-2079.

[4] Wassily Hoeffding. Probability inequalities for sums of bounded random variables. *J. Am. Stat. Assoc.*, 58(301):13–30, 1963. https://doi.org/10.1080/01621459.1963.10500830.
